# Graphene-based metasurface: dynamic optical control in ultrathin flat optics

**DOI:** 10.1515/nanoph-2025-0052

**Published:** 2025-04-22

**Authors:** Soojeong Baek, Hyeji Son, Hyunwoo Park, Hyeongi Park, Jaeyeong Lee, Sodam Jeong, Jae-Eon Shim, Jagang Park, Teun-Teun Kim

**Affiliations:** Department of Physics, 35029 University of Ulsan, Ulsan 44610, Republic of Korea; Department of Electrical and Computer Engineering, University of California, San Diego, CA 92093, USA; Department of Electrical Engineering and Computer Sciences, University of California, Berkeley, CA 94720, USA

**Keywords:** graphene-based metasurfaces, light–matter interaction, active optical devices, reconfigurable optical properties, active flat optics

## Abstract

Graphene hosts massless Dirac fermions owing to its linear electronic band structure. This distinctive feature underpins its extraordinary electronic properties, correlating to strong light–matter interactions on an extreme subwavelength scale. Over the past decade, intensive investigations have transitioned from fundamental graphene’s optical properties to practical application with the integration of graphene into metasurfaces, opening a new era of active flat optics. In this review, we provide a comprehensive overview of graphene-based metasurfaces, beginning with the intrinsic link between graphene’s optical response and its electronic properties. We highlight the development of actively tunable platforms and devices, including efficient modulators, high-sensitivity detectors, and advanced biosensing systems. We also discuss emerging approaches that enable ultrafast all-optical modulation and ultracompact device footprints, pushing the boundaries of performance. Finally, we explore the transformative prospects of non-Hermitian physics and inverse design strategies as novel frameworks for optimizing metasurface configurations. By synergizing graphene’s intrinsic tunability with innovative design methodologies, graphene-based metasurfaces hold immense potential to bridge the gap between fundamental science and real-world applications, defining a new frontier in next-generation photonic technologies.

## 1 Introduction

Graphene, a single atomic layer of carbon atoms arranged in a hexagonal lattice, exhibits exceptional electronic properties due to its unique linear energy-momentum dispersion near the Dirac points [1], [2]. This distinctive band structure enables charge carriers in graphene to behave as massless Dirac fermions, leading to ultrahigh carrier mobility, ballistic transport, and negligible effective mass. Beyond these electronic attributes, graphene also demonstrates remarkable optical characteristics, including broadband optical absorption, ultrafast carrier dynamics, and electrically tunable optical conductivity through gate-voltage modulation. Furthermore, recent advancements [3], [4], [5], [6], [7] in scalable fabrication techniques, particularly chemical vapor deposition (CVD), have enabled the production of high-quality, wafer-scale graphene with excellent uniformity and reproducibility. Such progress has enabled the transition of graphene from a material of fundamental interest to a practical platform for various technological applications [8], including optical modulator [9], [10], [11], [12], [13], [14], [15], [16], [17], transparent electrodes [18], [19], [20], [21], photodetectors [22], [23], [24], [25], light-emitting devices [26], [27], [28], [29], [30], photovoltaic cells [31], [32], radar [33], and adaptive thermal management [34].

Fundamentally, the atomically thin nature of graphene distinguishes it as a unique material for controlling light–matter interactions at the extreme subwavelength scale. Specifically, graphene’s optical response spans an exceptionally broad spectral range, from microwave to ultraviolet frequencies, governed predominantly by its complex surface conductivity. This conductivity can be dynamically modulated via electrostatic gating, optical excitation, or chemical doping, offering an effective route to tailor the interaction of photons with a one-atom-thick layer. In the near-infrared (NIR) and visible regimes, graphene exhibits universal absorption governed by interband transitions, while at terahertz (THz) and mid-infrared (MIR) frequencies, it behaves as a two-dimensional metal, supporting plasmonic excitations and enabling strong field confinement at deep-subwavelength scales. These intrinsic optical properties, coupled with its atomic-scale thickness (typically less than *λ*/100 at THz and MIR frequencies), provide an unparalleled platform for active and reconfigurable optical functionalities.

The integration of graphene with metasurfaces has opened a new avenue for active and reconfigurable control of electromagnetic waves at the extreme subwavelength scale. Metasurfaces [35] – planar arrays of subwavelength resonators – enable precise manipulation of the fundamental properties of light, such as amplitude, phase, and polarization within an ultrathin platform. However, conventional metasurfaces are inherently passive, lacking dynamic tunability once fabricated. Incorporating graphene into these architectures introduces an additional degree of freedom: real-time control of optical responses through external stimuli, including electrostatic gating and optical excitation. This hybridization leverages both the atomic-scale thickness and electrically tunable optical conductivity of graphene, facilitating ultrafast modulation, broadband tunability, and dynamic wavefront shaping. These capabilities surpass the limitations of passive photonic platforms, establishing graphene-based metasurfaces as a promising platform for next-generation optoelectronic devices, encompassing terahertz modulators, reconfigurable polarization optics, and nonvolatile photonic memory systems. Over the past decade, considerable efforts have been devoted to advancing the design, fabrication, and application of graphene-based metasurfaces, with emerging concepts such as programmable photonics, spatiotemporal modulation, and non-Hermitian physics further expanding their functional landscape.

**Figure 1:**
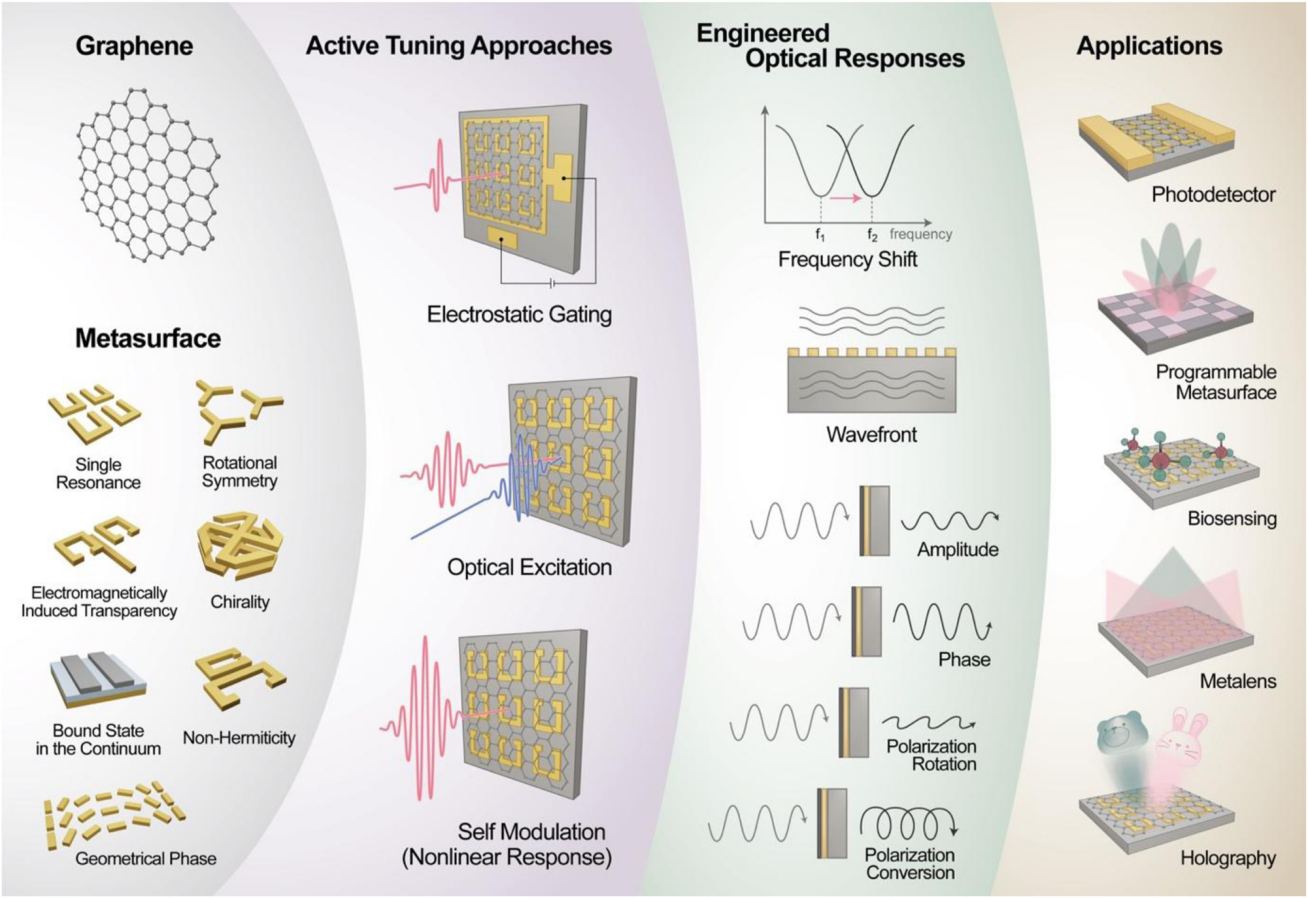
Conceptual illustration of graphene-integrated metasurfaces, highlighting representative metasurface designs (e.g., single resonance structures, chirality, bound states in the continuum, and geometrical phase elements) and various active tuning mechanisms including electrostatic gating, optical excitation, and nonlinear self-modulation. These active control methods allow precise engineering of optical responses such as frequency shifting, wavefront shaping, amplitude modulation, phase control, and polarization management. The combination of these capabilities facilitates diverse advanced applications ranging from photodetection and programmable optical devices to biosensing, metalenses, and dynamic holography.

In this review, we provide a comprehensive overview of graphene-based metasurfaces, focusing on their ability to achieve active optical control at extreme subwavelength scales. Figure 1 provides a conceptual overview of the key material properties of graphene, the underlying light–matter interaction mechanisms, and the functional integration of graphene with metasurfaces for active photonic applications. Section 2 explores the fundamental electronic and optical properties of graphene, highlighting its Fermi-level-dependent conductivity and resulting light absorption characteristics, as well as its plasmonic behavior. In Section 3, we introduce the design principles of graphene-based metasurfaces, achieved through the integration of electrically tunable graphene with functional metasurfaces. It further examines their functionalities in scattering, absorption, phase modulation, and wavefront shaping across THz, MIR, and NIR frequency ranges.

Section 4 introduces the latest advancements in graphene metasurface-based devices. Graphene’s linear and gapless band structure allows the intense electric fields of ultrashort laser pulses to generate high-energy electrons and holes within the material. This increases the density of charge carriers and temporarily enhances graphene’s conductivity. Section 4.1 discusses the development of intelligent and programmable graphene-based metasurfaces for real-time coding, adaptive optical manipulation, and neuromorphic functionalities. The demand for intelligent and programmable metasurfaces arises from the limitations of traditional static metasurface designs, which lack adaptability to dynamic environments and varying operational requirements. Modern optical and photonic systems increasingly require real-time control over wavefront shaping, scattering, and polarization to enable applications in areas such as adaptive optics, wireless communication, and artificial intelligence. Section 4.2 focuses on biosensing applications that leverage graphene’s high sensitivity and dynamic tunability for molecular detection. Biosensing applications are essential to meet the growing demand for sensitive, rapid, and accurate detection in healthcare, environmental monitoring, and biochemical research. Conventional sensors often struggle with low sensitivity and adaptability in complex environments. Graphene’s unique properties, such as its high surface area, electrical conductivity, and tunable optical characteristics, make it ideal for amplifying detection signals and enabling real-time molecular monitoring. Its dynamic tunability further allows for versatile, multifunctional biosensors capable of detecting a wide range of targets. Section 4.3 examines the integration of graphene-based metasurfaces in photodetectors. Various optical devices utilize graphene’s photoexcitation effect, including high-speed photodetectors, polarization sensors, and multidimensional optical processors. The interaction between graphene and structured optical fields enhances photodetection efficiency, polarization selectivity, and spectral tunability, paving the way for next-generation optoelectronic devices.

In Section 5, we address the practical challenges associated with the scalable fabrication of high-quality graphene-based metasurfaces. This includes a comprehensive discussion on the growth and transfer processes of graphene, highlighting key issues such as structural defects, surface contamination, and uniformity degradation. Recent advances in substrate engineering and transfer techniques are introduced as essential strategies to enable reliable, wafer-scale integration of graphene-based metasurfaces for real-world applications.

Section 6 provides an outlook on advanced modulation approaches and design strategies for graphene-based metasurfaces, aimed at enhancing their performance, scalability, and potential in next-generation photonic applications. Section 6.1 explores nonlinear optical modulation and all-optical control methods aimed at achieving ultrafast modulation and signal processing, which are essential for next-generation optical devices. Section 6.2 provides an overview of emerging strategies such as non-Hermitian metasurfaces incorporating gain and loss, concluding with a discussion on integrating computational inverse design techniques to enhance device performance and scalability. By synthesizing recent progress and outlining future opportunities, this review aims to provide a strategic perspective on graphene-based metasurfaces, underscoring their potential to revolutionize photonic technologies for sensing, communication, and quantum applications.

## 2 Fundamentals of graphene

### 2.1 Electronic properties of graphene

**Figure 2:**
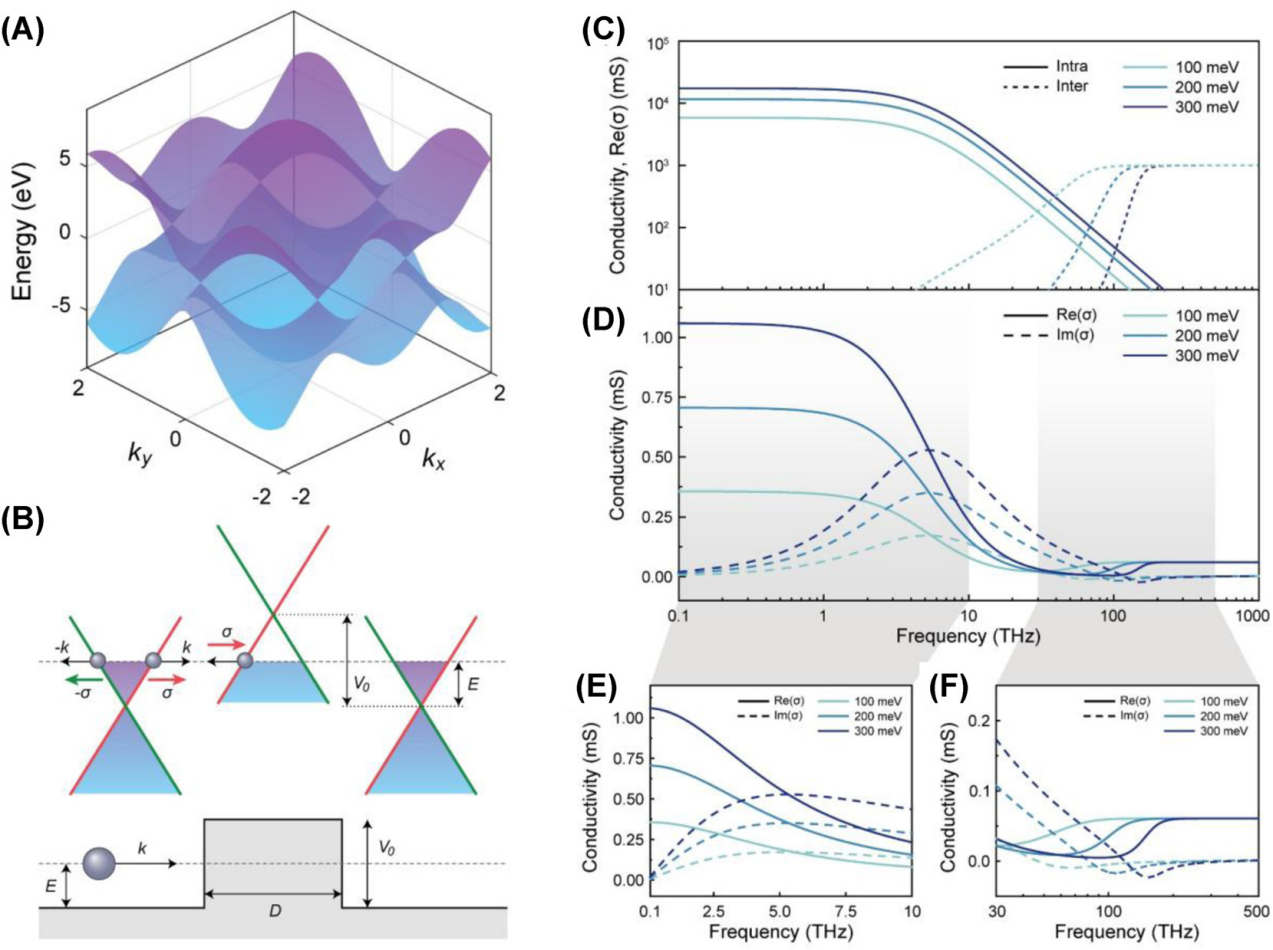
Electronic and optical properties of graphene from THz to visible range. (A) Graphene band structure calculated using the tight-binding approximation. (B) Illustration of Klein tunneling in graphene. (C) Intraband and interband contributions to the optical conductivity of graphene at different Fermi levels. (D) Total optical conductivity spectrum of graphene, combining intraband and interband effects. (E) Optical conductivity in the THz–MIR range, showing increasing conductivity with Fermi level due to enhanced Drude response. (F) Optical conductivity near the NIR and visible range, exhibiting a universal value beyond the onset frequency of interband transitions.

Graphene, often modeled using the tight-binding approximation as shown in Figure 2A, is classified as a zero-gap semiconductor due to the touching of its valence and conduction bands at the Dirac points (K and K′) in the first Brillouin zone [1], [2], [36]. Near these Dirac points, the energy-momentum relationship becomes linear, $E_{k}=\pm v_{F}\left|k\right|$ , where *v*_*F*_ is the Fermi velocity, approximately *c*/300 (*c*: speed of light, [2]). This linear dispersion causes the charge carriers in graphene – both electrons and holes – to behave as massless Dirac fermions, exhibiting properties fundamentally distinct from conventional Schrödinger fermions with quadratic energy dispersion. The relativistic-like dynamics of graphene’s quasiparticles give rise to a range of unique physical phenomena. Notable examples include the Klein paradox (Figure 2B, [37]), which describes the unusual ability of massless particles to tunnel through high potential barriers, and chiral tunneling, where the charge carriers’ pseudospin aligns with their direction of motion, preventing backscattering. Furthermore, graphene demonstrates the integer quantum Hall effect at room temperature, a remarkable property arising from its robust quantum conductivity quantization even under moderate external magnetic fields [38]. Graphene’s thermal and electrical properties are equally exceptional, with electron mobilities reaching up to 10^5^ Cm^2^/Vs at room temperature, enabling ballistic transport over micrometer scales. Additionally, graphene maintains minimal conductivity, *σ*_min_ = 4*e* ^2^/*h*, even in the absence of charge carriers, an attribute stemming from its gapless electronic structure [39], [40]. These unique electronic properties position graphene as a versatile material for a broad range of applications, particularly in high-speed electronics, quantum transport devices, and energy-efficient systems, where its ultrafast charge carrier dynamics and exceptional mobility can be fully exploited.

### 2.2 Light–matter interaction in graphene

The distinctive linear band structure of graphene not only governs its electronic properties but also leads to unique optical behavior. Specifically, graphene’s interaction with light can be categorized into two distinct regimes – intraband and interband transitions – depending on the photon energy (*ℏω*) relative to the Fermi level (*E*_*F*_) as in Figure 2C–F [41], [42], [43], [44], [45], [46], [47], [48]. When the photon energy exceeds twice the Fermi level ( $\hbar \omega >2\left|E_{F}\right|$ ), interband transitions dominate, where electrons are excited across the Dirac cone. This process leads to graphene’s universal absorption of incident light, approximately *πα* = 2.3 % across a broad frequency range. Here, *α* is the fine-structure constant. This universal absorption arises from graphene’s linear dispersion and is described by the interband optical conductivity:

(1) $$\sigma_{\text{inter}}\left(\omega \right)=\frac{e^{2}}{4\hbar }\left[\tan h\left(\frac{\hbar \omega +2E_{F}}{4k_{B}T_{e}}\right)+\tan h\left(\frac{\hbar \omega -2E_{F}}{4k_{B}T_{e}}\right)\right],$$

where *k*_*B*_ is the Boltzmann constant, and *T*_*e*_ is the electron temperature. For high photon energies ( $\hbar \omega \gg 2\left|E_{F}\right|$ ), the conductivity simplifies to the universal value $\sigma_{\text{inter}}=\frac{e^{2}}{4\hbar }$ . In contrast, for photon energies below $2\left|E_{F}\right|$ , interband transitions are suppressed by Pauli blocked, and intraband transitions become dominate. In this regime, graphene exhibits a Drude-like free-carrier response, with its conductivity expressed as:

(2) $$\sigma_{\text{intra}}\left(\omega \right)=\frac{e^{2}}{\pi \hbar }\frac{k_{B}T_{e}}{E_{F}}\frac{\tau }{1-i\omega \tau }\ln\left[2\cos h\left(\frac{E_{F}}{2k_{B}T_{e}}\right)\right],$$

where *τ* is the carrier momentum scattering time. In the low temperature limit (*T*_*e*_ ≪ *E*_*F*_/*k*_*B*_), this expression simplifies to:

(3) $$\sigma_{\text{intra}}\left(\omega \right)\approx \frac{e^{2}E_{F}}{\pi \hbar }\frac{\tau }{1-i\omega \tau }.$$

This Drude conductivity governs graphene’s optical behavior in the THz and MIR frequency ranges [41].

To clarify these two regimes, Figure 2C separately depicts the spectral dependence of intraband and interband contributions. The total optical conductivity spectrum, combining both effects, is presented in Figure 2D, which clearly illustrates the crossover region around ℏω ≈ 2|*E*_*F*_| – a critical point where the optical response is mainly governed by both transitions.

For practical applications of graphene-based metasurfaces, it is important to distinguish which optical regime is utilized. Figure 2E and F highlights these operational windows. In Figure 2E, corresponding to the THz and MIR ranges, the intraband regime is emphasized. Here, the real and imaginary parts of the Drude conductivity increase with *E*_*F*_, enabling graphene to mimic metallic behavior despite its atomic-scale thickness. This tunable free-carrier response underpins key functionalities such as amplitude and phase modulation in THz graphene-based metasurfaces. In contrast, Figure 2F focuses on the interband regime near the NIR and visible ranges. The onset frequency of interband transitions shifts with *E*_*F*_, but beyond the threshold, graphene’s optical conductivity converges to the universal value, independent of gating. This spectral characteristic has been leveraged for applications including tunable absorption and light modulation in graphene-based metasurfaces at higher frequencies. By explicitly illustrating the distinct spectral regimes and corresponding conductivity characteristics, we clarify under what conditions each contribution governs device operation. This understanding is essential for the strategic design of electrically tunable metasurfaces and light–matter interaction devices based on graphene, across an exceptionally broad spectral range – from THz to visible frequencies.

## 3 Graphene-based metasurface for active flat optics

Recent studies have demonstrated that single-layer graphene, despite its atomic thickness, can exhibit substantial optical modulation, particularly in the THz and microwave frequency regimes. In these spectral ranges, the high carrier mobility and tunable optical conductivity of graphene have enabled effective modulation of both the amplitude and phase of incident waves without the need for additional nanostructures or resonant elements. Representative examples include the works [33], [49], [50], [51], [52], which have demonstrated substantial modulation depths and practical device performances based on reflective resonant structures incorporating graphene thin films. However, in transmissive device configurations and at higher frequency ranges, the atomically thin nature of graphene often results in inherently weak light–matter interactions. To overcome these limitations and further broaden the functional scope of graphene-based devices, significant efforts have been directed toward integrating graphene with metasurfaces. This hybrid approach not only enhances interaction strength by leveraging localized resonances and engineered electromagnetic responses but also introduces additional degrees of freedom to enable multifunctionality, including broadband operation, dynamic beam shaping, polarization control, and complex wavefront engineering. In this section, we review key studies that harness the synergistic combination of graphene and metasurfaces to realize advanced, multifunctional optical capabilities beyond those achievable with graphene alone.

### 3.1 Electrically tunable graphene-based metasurfaces

#### 3.1.1 Graphene-based metasurface for dynamic scattering and absorption control

Electrically tunable graphene-based metasurfaces exploit the carrier-density-dependent optical conductivity of graphene [39], [40], [41], [53], which can be dynamically modulated via integrated gating schemes [54], [55], [56]. By embedding graphene into passive metasurface architectures, researchers have demonstrated real-time control over electromagnetic responses across various configurations, including transmissive planar geometries, reflective resonators, and perfect absorbers. These designs typically utilize engineered resonant structures to enhance light–matter interactions and enable dynamic modulation of amplitude, phase, and polarization. Such hybrid platforms offer versatile optical functionalities, including tunable transmission, reflection, absorption, wavefront shaping, and polarization manipulation, thereby addressing complex photonic applications such as reconfigurable beam steering and adaptive spectral filtering. Notably, the dominant light–matter interaction mechanisms – whether intraband or interband transitions – vary across spectral regions, leading to distinct modulation behaviors. This characteristic has guided the development of graphene-based metasurfaces tailored to specific spectral ranges. In the following subsections, we review representative studies categorized by their operational regimes in the NIR, MIR, and THz frequencies. We highlight how the synergy between graphene’s tunability and various multifunctional metasurface designs has enabled dynamic, broadband, and efficient optical modulation.

**Figure 3:**
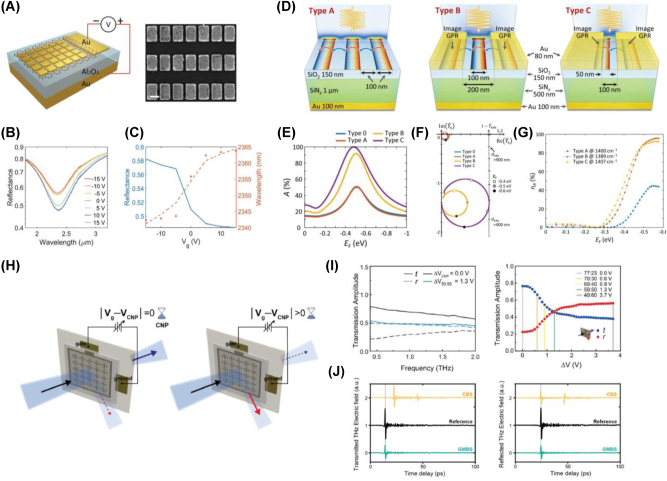
Dynamically tunable absorption and beam splitting. (A) Schematic representation of the hybrid graphene-based metasurface device, combining metallic plasmonic structures (left), and SEM image of the fabricated sample (right). (B) Measured reflectance spectra of the metasurface with different gate voltages. (C) Measured reflectance at 2.42 µm (blue curve) and resonant wavelength (orange dots) under different gate voltages, demonstrating stable reflectance changes with a modulation depth of up to 17 % [57] (Copyright 2022, John Wiley and Sons). (D) Schematic of type A, B, and C graphene plasmonic structures designed for perfect absorption. (E) Absorption maps of type A, B, and C structures showing tunable absorption with varying graphene Fermi levels, demonstrating dynamic control of resonant absorption frequencies. (F) Surface admittance charts illustrating the admittance matching conditions for type A, B, and C structures, with type B and C demonstrating superior absorption efficiency through enhanced coupling to free space. (G) Experimentally measured absorption and modulation efficiency at the frequency for maximum absorption in each structure, demonstrating near-perfect absorption with type B [67] (Copyright 2018, American Chemical Society). (H) Schematic representation of the graphene-based metasurface beam splitter. (I) (left) Transmission, reflectance, and (right) splitting ratio of the graphene-based metasurface beam splitter as a function of gate voltage, demonstrating tunable THz beam splitting across a broad frequency range at low gate voltage levels. (J) Experimental THz waveforms of transmitted and reflected beams for graphene-based metasurface beam splitter and commercially available beam splitter [77] (Copyright 2023, Optica Publishing Group).

In the NIR regime, interband transitions dominate, resulting in a constant absorption as photons excite electrons from the valence band to the conduction band. Consequently, graphene is primarily employed as a transparent electrode in this spectral range. To overcome these limitations, structures incorporating a single-layer graphene with a gold back reflector and a 600 nm-thick silica spacer layer, or combined with plasmonic nanostructures exhibiting Fano resonances, have been employed [55], [57], [58]. However, these approaches were constrained by a very low tuning range (≤5 %). Recently, Cai et al. [57] achieved a modulation depth of approximately 17 % at relatively low voltages by combining single-layer graphene with metallic plasmonic structures and introducing an Al_2_O_3_ spacer layer, thus enabling a “step-like” change in reflectance driven by interband transitions (Figure 3A–C). Due to its low dependence on graphene’s carrier mobility, this approach ensures high stability even under experimental conditions.

In contrast, in the MIR region [59], [60], [61], [62], [63], [64], [65], the optical response of graphene is influenced not only by interband transitions but also by intraband transitions – electron dynamics within the same band – and by plasmonic resonances. Because MIR photons have much lower energy than those in the visible or NIR range, it becomes relatively straightforward to adjust the occupancy of specific energy states by shifting the Fermi level. This ability enables active tuning of the optical response through control of the plasmon resonance frequency. Moreover, in this spectral range, real-time tuning is achievable via applied gate voltages, paving the way for advanced applications in optical modulators, switches, and infrared sensors. Experimental demonstrations have shown that these principles offer high tunability of light–matter interactions in this spectral range [66]. For instance, by employing surface admittance matching and localized plasmonic effects, researchers have realized electronically tunable perfect absorption. These hybrid metasurfaces integrate graphene with metallic nanostrips, thereby concentrating electromagnetic fields and matching the system’s admittance to that of free space (Figure 3D–G, [67]). As a result, near-perfect absorption occurs at specific Fermi levels, and the absorption peaks shift dynamically under electrical gating (Figure 3E). This capability enables modulation efficiencies approaching 100 % (Figure 3G), making the technology well-suited for advanced applications in thermal management and infrared sensing.

As we move into the THz regime [15], [16], [68], [69], [70], [71], [72], [73], [74], [75], [76], the electrical response of electrons themselves becomes more pronounced than collective electron motion (plasmons) or electron–hole pair generation in graphene. In this regime, intraband conductivity predominantly governs the optical response, and the Fermi level can be substantially altered through doping or gate voltage modulation [16]. This allows for active control over reflection, transmission, and phase changes. Not only can amplitude and phase modulation be realized by applying resonant effects, but by designing the resonant frequency to lie outside the frequency band of interest and employing frequency-insensitive, nondispersive responses, it is also possible to achieve greater modulation over a wider operational frequency range [74], [77]. The metasurface [77], illustrated in Figure 3H, combines graphene with metallic meta-atoms to achieve consistent transmission and reflection across the 0.5–1.5 THz range (Figure 3I). This system achieves a precise 50:50 splitting ratio at low gate voltages, and it was shown that the transmission and reflection ratios can be tuned from 77:23 to 40:60 by varying the gate voltage. Additionally, it is fabricated on an ultrathin polymer substrate that is negligible compared to the central wavelength of the incident THz pulse, preventing the observation of Fabry–Pérot etalon effects in the transmitted and reflected THz waveforms (Figure 3J). The absence of spectral interference ensures compatibility with high-precision spectroscopy and imaging systems, enhancing signal clarity for sensitive measurements.

These examples demonstrate how graphene’s tunable intraband and interband transitions, combined with metasurface design, enable precise control over dynamic scattering and absorption. Leveraging these properties, graphene-based metasurfaces offer compact, interference-free, and broadband solutions for next-generation photonic technologies.

#### 3.1.2 Electrical control of phase, wavefront engineering, and polarization

Reconfigurable phase control represents a fundamental capability in modern photonics, enabling key functions such as beam steering, focusing, and polarization manipulation. Beyond merely guiding the propagation of light, dynamic phase modulation serves as the foundation for multifunctional photonic devices, supporting advanced applications in sensing, imaging, holography, and high-speed communication. In this context, graphene-based metasurfaces have emerged as an effective platform for electrically tunable phase control, owing to the gate-dependent optical conductivity of graphene. By carefully tailoring critical coupling conditions and exploiting modal interactions – including loss-controlled critical coupling and avoided mode crossing – these systems achieve efficient and real-time phase modulation. These capabilities not only facilitate dynamic wavefront shaping but also open new avenues for the development of compact, multifunctional photonic devices.

**Figure 4:**
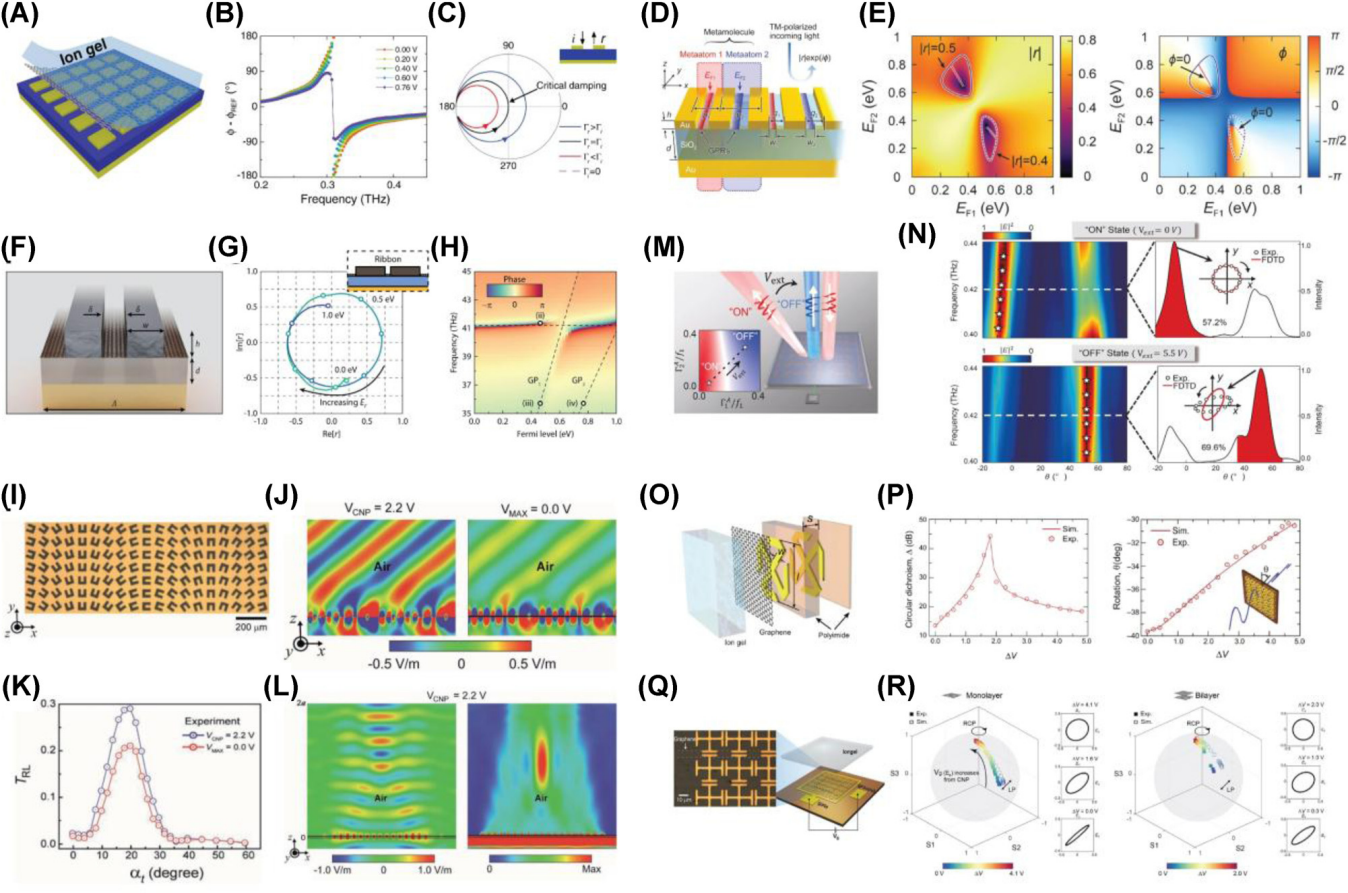
Dynamic control of phase, polarization, and beam steering. (A) Schematic of the graphene-based metasurface integrating a one-port resonator design. (B) Gate-dependent phase spectra showing the transition from underdamped to overdamped regimes through critical damping. (C) Smith curves illustrating the evolution of the reflection coefficient, showing critical damping between underdamped and overdamped behavior [78] (Copyright 2015, American Physical Society). (D) Schematic of the graphene plasmonic metamolecule designed for mid-infrared phase and amplitude modulation. (E) Amplitude (left) and phase (right) maps of the reflection coefficient, showing dual-parametric control via independent Fermi-level tuning of the graphene plasmonic ribbons [66] (Copyright 2020, American Chemical Society). (F) Schematic of the graphene-based metasurface designed to achieve phase modulation. (G) Complex reflection coefficients as the Fermi level is tuned from 0 to 1 eV. (H) Plot of the frequency dependence of the reflected phase, derived from the reflection coefficients shown in (G) [79] (Copyright 2022, Springer Nature). (I) Fabricated supercell image of graphene-based metasurfaces for electrically tunable anomalous refraction. (J) Simulated electric field distribution of THz waves at two gate voltages (2.2 V and 0 V). (K) Measured cross-polarized transmission intensity at 1.15 THz as a function of refraction angle with two gate voltages. (L) Simulated electric field (left) and energy density distributions (right) at 1.15 THz [83] (Copyright 2017, John Wiley and Sons). (M) Schematic of a gate-tuned graphene-based metasurface designed for dynamic THz beam steering. By adjusting the gate voltage, the metasurface can switch between ON (anomalous reflection) and OFF (normal reflection) states, leveraging critical coupling and PB phase gradients. (N) Experimental demonstration of ON/OFF switching in a gate-tuned graphene-based metasurface with an incident angle of −50°. The left panel shows the scattered electric field intensity of the reflected wave, while the right panel illustrates the deflection efficiency. ON state shows anomalous reflection at −7.3°, and OFF state exhibits normal reflection at 50° [84] (Copyright 2022, De Gruyter). (O) Schematic of a gate-controlled graphene-based chiral metamaterial. (P) Gate-controlled circular dichroism at the resonance frequency (left) and optical activity at an off-resonance frequency (right) [85] (Copyright 2017, The American Association for the Advancement of Science). (Q) Schematic of the graphene-based metasurfaces for electrically tunable polarization control and microscopic image. (R) Experimental and simulated polarization state evolution on the Poincaré sphere, showing voltage-dependent polarization control with mono- (left) and bilayer (right) graphene-based metasurfaces [86] (Copyright 2023, De Gruyter).

In the THz regime, incorporating graphene into reflective metasurfaces provides a precise route to phase manipulation via gate voltages [78] in illustrated in Figure 4A–C. By tuning graphene’s conductivity, the system transitions from underdamped to overdamped behavior, reaching a critical coupling condition where intrinsic and radiative losses are balanced. Under these conditions, a full 2*π* phase shift can be achieved through the avoided crossing between graphene plasmonic resonances and the cavity mode. These loss-mediated dynamics illustrate the potential of graphene for efficient THz phase modulation. In the MIR regime, graphene-based metasurfaces further extend the scope of phase modulation. For instance, integrating two independently gated graphene plasmonic ribbons with metallic antennas (Figure 4D) enables a complete 2*π* phase shift via precise Fermi-level tuning (Figure 4E, [66]). This configuration exploits strong light–matter interactions to achieve beam steering and wavefront shaping without relying on additional dispersive components. Geometric optimization and mode engineering enhance plasmonic coupling, facilitating efficient phase control and dynamic modulation of optical fields. More recently, quasi-bound states in the continuum (qBIC) have been explored as an alternative strategy for phase modulation [79]. In this approach, the interplay between graphene’s tunable conductivity and avoided crossing phenomena enables phase shifts exceeding 3*π*, with a theoretical limit approaching 4*π* (Figure 4F–H). Such advancements highlight the versatility of graphene-based metasurfaces, which enable phase modulation beyond 3*π*, a notable increase compared to conventional designs typically limited to ∼2*π*.

By leveraging the spatial phase delay distribution of metasurfaces [80], [81], [82] and the unique tunability of graphene’s electronic properties, researchers have established a robust technological foundation for a wide range of applications, including beam steering and holography. One notable demonstration [83] involves actively modulating the amplitude of anomalously refracted THz waves (Figure 4I–L). In this approach, U-shaped metallic apertures integrated with a graphene monolayer are arranged to generate a spatially varying Pancharatnam–Berry (PB) phase profile. By applying an electrical gate to the graphene and thus its corresponding optical conductivity can be adjusted, achieving a modulation depth of 28 % for cross-polarized THz waves at 1.15 THz. Furthermore, implementing a parabolic PB phase distribution enables focusing of THz beam and allows for active amplitude and wavefront control without mechanical adjustments. Building on these principles, Li et al. demonstrate a gate-tunable graphene-based metasurface capable of switching between normal and anomalous reflection modes by combining critical coupling with PB phases (Figure 4M and N, [84]). In this reflective configuration, critical coupling ensures a delicate balance between intrinsic and radiative losses, while the PB phase gradient dictates the direction of the reflected beam. By tuning graphene’s conductivity, the system transitions seamlessly between regimes of dominant intrinsic loss (yielding anomalous reflection) and dominant radiative loss (yielding normal reflection). This on/off switching paradigm optimizes energy efficiency by eliminating transmission pathways, representing a significant advancement in THz photonics for agile beam steering, sensing, and holographic functionalities.

Active control over polarization states is equally vital in cutting-edge photonic systems, and graphene-based metasurfaces have demonstrated considerable promise for such dynamic polarization manipulation. One notable approach [85] employs chiral metamolecule structures hybridized with gated monolayer graphene (Figure 4O). Through strategic gating, the intrinsic loss within the system can be modulated, enabling precise control of circular dichroism and optical activity. By navigating through radiative, intrinsic, and critical coupling regimes, the metamolecules achieve remarkable circular dichroism of up to 45 dB. Off-resonance, polarization rotations as large as 10° have been measured for linearly polarized THz waves, representing a noteworthy capability to manipulate polarization states at room temperature without magnetic fields (Figure 4P). More recently, Park et al. [86] proposed a structure that combines metasurfaces with monolayer and bilayer graphene to achieve refined polarization control in the THz domain (Figure 4Q). In this design, graphene layers are placed between capacitive meta-atoms, and the resulting conductivity changes modulate the metasurface’s capacitive coupling. With monolayer graphene, a phase delay of up to 81° can be achieved, while bilayer graphene significantly reduces the voltage required to realize a full 90° phase delay (Figure 4R). This system functions as an electrically tunable THz quarter-wave plate, underscoring the potential of graphene-based metasurfaces for versatile and efficient polarization control.

#### 3.1.3 Comparison of graphene-based metasurfaces with other active materials platforms

A variety of active materials have been integrated into metasurfaces to achieve dynamic control of their optical response [87], [88], [89], [90], [91], [92], [93], [94], [95], [96], including semiconductors, transparent conductive oxides (TCOs), phase-change materials (PCMs), liquid crystals (LCs), and electro-optic polymers. Each material platform possesses distinct merits and limitations, determined by their fundamental tuning mechanisms and material properties.

Semiconductors and TCOs primarily exploit free carrier density modulation to achieve dynamic tunability, enabling a moderate refractive index change in the range of Δ*n* ≈ 0.08–1.5 with fast modulation speeds up to the MHz regime and beyond. Specifically, semiconductor platforms such as silicon and III–V compounds (e.g., GaAs, InP) typically exhibit Δ*n* ≈ 0.08 at a wavelength of 1.5 μm under carrier injection, while TCOs, exemplified by indium tin oxide (ITO), demonstrate a larger index modulation of Δ*n* ≈ 1.5 with electrical modulation speeds reaching up to 10 MHz. This approach benefits from compatibility with mature semiconductor processing technologies and offers reliable electrical tunability, although the index modulation is typically accompanied by increased optical losses due to free carrier absorption. Phase-change materials, such as VO_2_ and chalcogenides, exhibit large, nonvolatile refractive index contrast (Δ*n* ≈ 0.5–3) arising from thermally driven structural phase transitions. For example, VO_2_-based metasurfaces show Δ*n* ≈ 0.5 and chalcogenide PCMs reach Δ*n* ≈ 2.8. Their switching behavior is robust and retains the optical state without external power, making them advantageous for memory and display applications. However, PCM-based metasurfaces suffer from relatively slow response times (∼kHz–MHz) and limited endurance, typically around 10^8^ cycles. Liquid crystals and electro-optic polymers offer low-loss, volatile tunability with moderate index changes (Δ*n* ≈ 0.01–0.1). Typical LC-based metasurfaces achieve Δ*n* ≈ 0.1 with response speeds in the 100 Hz–kHz range. These platforms have been widely employed in dynamic beam steering and display technologies, but their tuning speed is inherently limited by molecular reorientation dynamics, and they suffer from temperature-dependent performance.

Graphene-based metasurfaces [97], [98] offer an alternative platform with distinctive characteristics. The key advantage of graphene arises from its atomic thickness and linear band dispersion near the Dirac point, which leads to an exceptionally strong modulation of its optical conductivity with relatively small carrier injection. Experimentally, graphene-based metasurfaces have demonstrated a refractive index change up to Δ*n* ≈ 1 in the terahertz regime, with fast, continuous, and reversible electrical tunability of the plasmonic and optical properties without requiring structural phase changes or mechanical deformation. Graphene also supports ultra-confined plasmonic modes and enables modulation speeds exceeding 30 MHz, with endurance over 10^9^ cycles. These speeds are fundamentally limited not by the material itself but by the device architecture. Moreover, graphene’s compatibility with planar integration and the possibility of electrical gating at the meta-atom level further enhance its appeal for on-chip photonic applications. However, the tuning range of graphene-based metasurfaces is generally limited to the MIR and THz regimes, as a consequence of the plasmon dispersion scaling with Fermi energy and geometrical size. Pushing the operation toward visible or NIR wavelengths requires either extreme electrostatic doping or sub-10 nm patterning, which is technologically challenging. Additionally, similar to TCOs and semiconductors, graphene-based devices inherently exhibit increased optical loss during index modulation, stemming from free carrier absorption.

## 4 Application of graphene-based metasurface

### 4.1 Programmable and intelligent graphene-based metasurface

The on-demand tunability of graphene facilitates the development of intelligent metasurfaces that can dynamically adapt their functionalities in real-time [99], [100], [101], [102]. This section explores the advancements in coding and programmable graphene-based metasurfaces.

**Figure 5:**
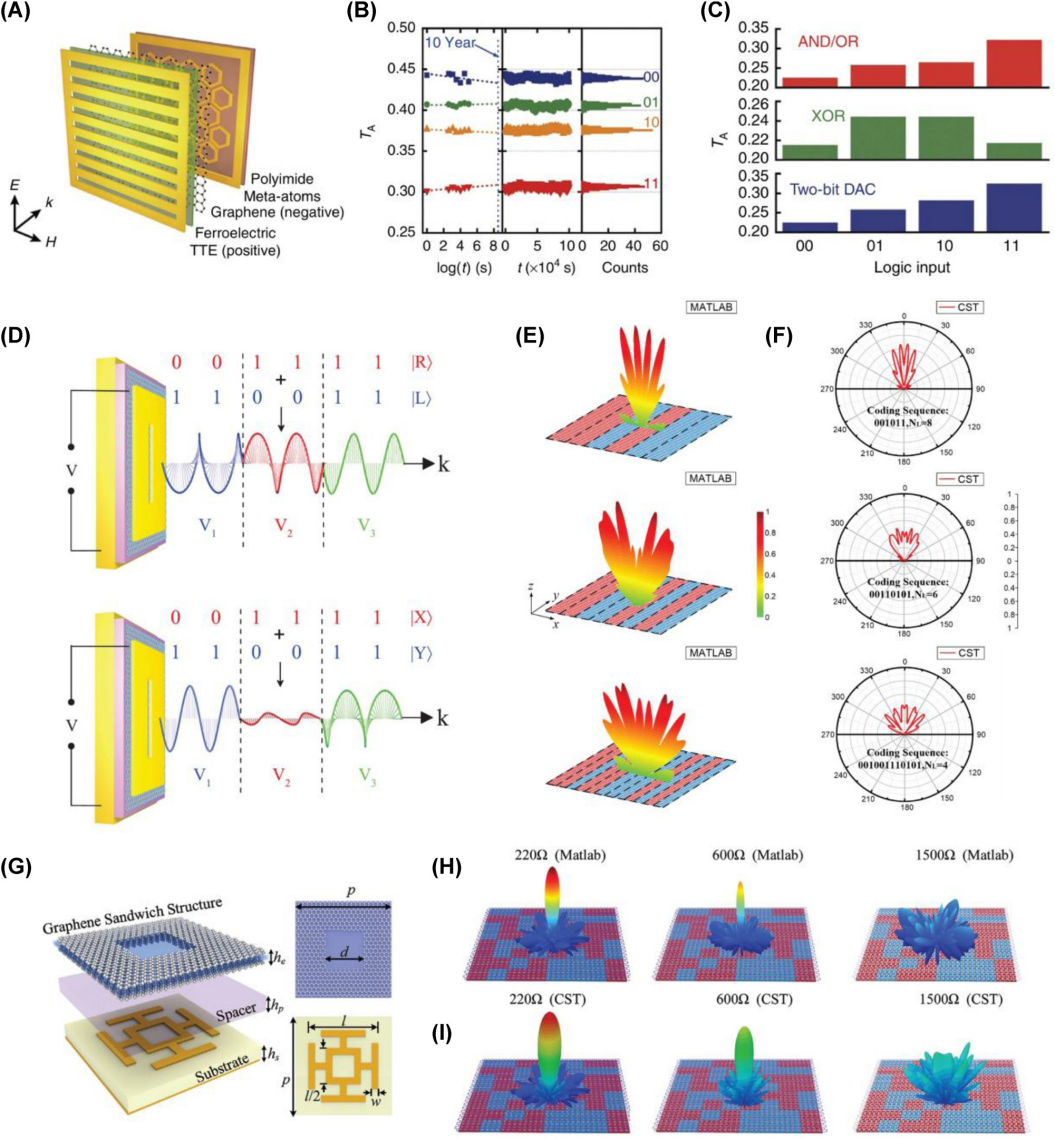
Nonvolatile memory, logic operations, and programmable electromagnetic responses. (A) Schematic representation of the graphene-ferroelectric metadevice designed for nonvolatile memory retention. (B) Retention performance of the graphene-ferroelectric memory device over time. (C) Measured transmission amplitudes for logic operations, demonstrating the graphene-ferroelectric metadevice’s capability for AND, OR, and XOR functionalities [103] (Copyright 2016, Springer Nature). (D) Schematic illustration of realizing the PDM technique by the proposed metasurface with the circular (top) and linear (bottom) orthogonal polarization basis according to the superposition principle of polarization states [104] (Copyright 2015, John Wiley and Sons). (E) Analytically calculated and (F) numerically simulated results of far-field scattering patterns for the microwave programmable graphene metasurface (MPGM) under various coding sequences, demonstrating enhanced scattering diversity and effective radar cross section (RCS) reduction [99] (Copyright 2020, American Chemical Society). (G) Schematic and design details of the proposed graphene-based coding metasurface. (H) Calculated and (I) simulated far-field scattering patterns of the proposed metasurface under varying sheet resistance values, illustrating the transition from specular reflection to diffuse scattering [101] (Copyright 2020, John Wiley and Sons).

One of the most promising applications of graphene-based metasurfaces is in memory and logic gate technologies. To support advanced coding schemes, it is essential to establish robust memory capabilities at the fundamental hardware. The integration of graphene with ferroelectric materials has driven notable progress in this area [103]. Graphene-ferroelectric metadevices demonstrate multistate, nonvolatile memory retention exceeding 10 years at ambient temperatures without requiring continuous external stimuli (Figure 5A and B). Furthermore, they can perform complex logic operations including AND, OR, and XOR gates, highlighting their potential for compact and scalable computing architectures (Figure 5C).

Beyond fundamental memory and logic functionalities, graphene-based metasurfaces present promising avenues for optical signal processing and neuromorphic computing. Building on these foundational memory and logic gate capabilities, they extend their functionality to sophisticated coding applications in the optical and THz domains. For instance, Li et al. [104] demonstrated polarization control and optical encoding in the MIR range using graphene-loaded plasmonic metasurfaces, achieving switching speeds of up to 40 MHz. By varying the applied gate voltage, continuous polarization modulation from linear to circular was realized. This precise control enables polarization-division multiplexing, enhancing data transmission capacity within a single channel (Figure 5D).

Programmable graphene metasurfaces further expand system capabilities by enabling dynamic, real-time control over electromagnetic wave properties [99]. Chen et al. exploited graphene’s electrical tunability to independently manipulate the amplitude and phase of reflected signals. By combining amplitude modulation with phase coding, they achieved reconfigurable scattering patterns in real-time. A notable demonstration involved switching from mirror-like reflection to diffuse scattering, underscoring the potential of graphene metasurfaces for applications such as radar cross section (RCS) reduction (Figure 5E and F). Dynamic scattering steering marks another advancement achieved through graphene-based metasurfaces. Zhang et al. [101] utilized graphene’s resistive properties to realize binary coding elements with opposite phases and uniform amplitudes. By implementing a 1-bit coding metasurface, they demonstrated modulated scattering patterns (Figure 5G–I), further illustrating the versatility and effectiveness of graphene-based solutions for controlling electromagnetic wave propagation.

These developments illustrate the transformative potential of graphene-based metasurfaces in various advanced applications, from memory and logic operations to dynamic wavefront control and signal processing. As research progresses, the integration of graphene with other materials and the refinement of coding techniques will further enhance the capabilities and applications of intelligent graphene-based metasurfaces.

### 4.2 Biosensing with graphene-based metasurface

Graphene has emerged as an attractive material for biosensing applications due to its unique combination of electrical, optical, and chemical properties. Its atomic-scale thickness ensures a large surface-to-volume ratio, promoting effective interaction with biomolecules. Moreover, graphene’s gate-tunable conductivity, high carrier mobility, and strong near-field enhancement capabilities enable sensitive detection of molecular species. Building on these attributes, various studies [105], [106], [107], [108], [109], [110] have demonstrated graphene-based metasurface biosensors operating across the terahertz (THz) and mid-infrared (MIR) regimes, targeting applications such as molecular detection, environmental monitoring, and food safety. Graphene’s integration with metasurfaces has facilitated dynamic, label-free biosensing with enhanced sensitivity and spectral selectivity.

**Figure 6:**
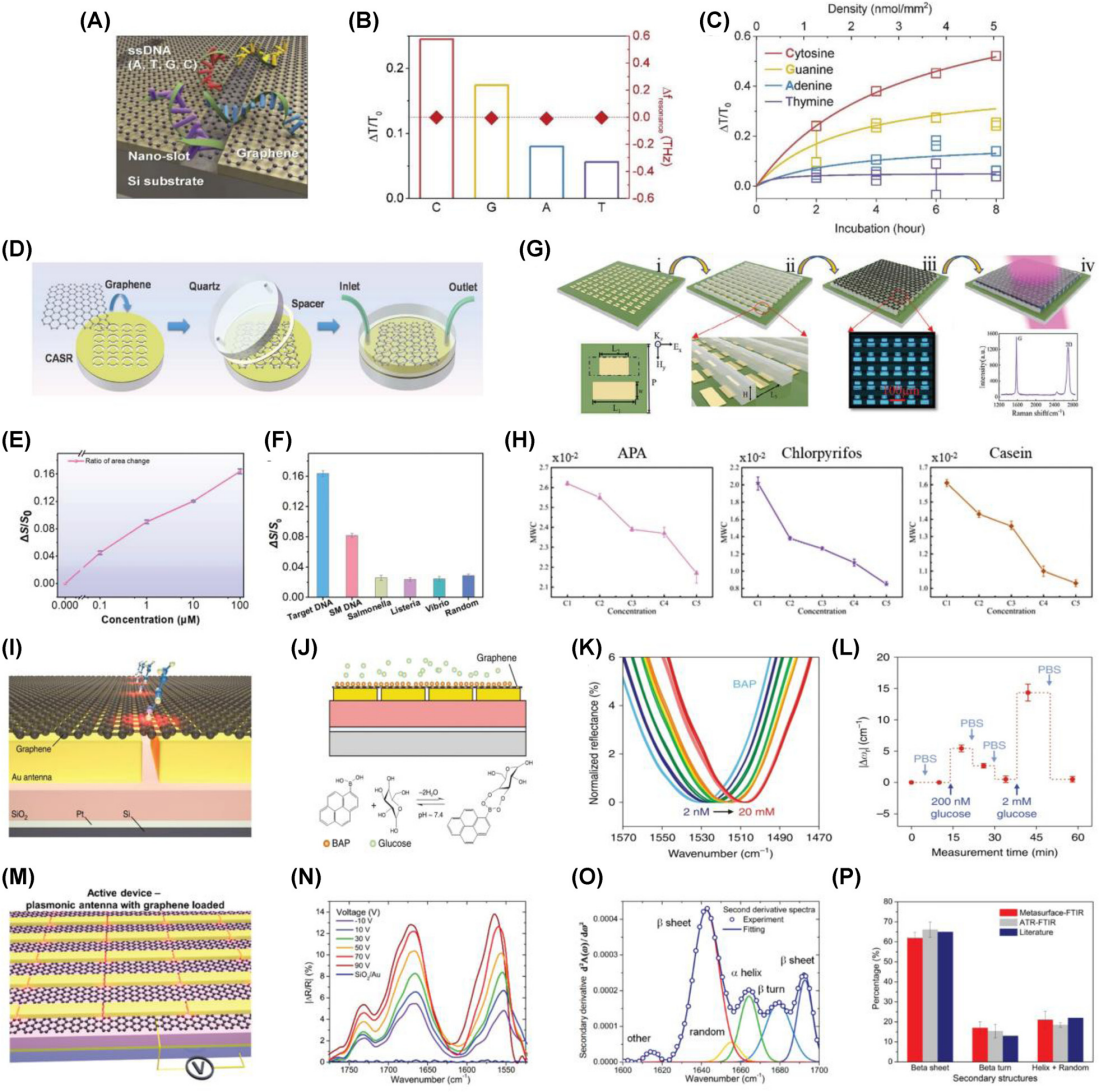
Highly sensitive and selective detection. (A) Schematic of the graphene-combined nanoslot metamaterial for ultra-sensitive sensing of DNA. (B) Transmission change and resonance shift of the graphene-covered nanoslot with ssDNA adsorption. (C) Monitored THz transmittance of the graphene-combined nanoslot metamaterial according to the incubation time and corresponding density of the ssDNA [105] (Copyright 2020, Elsevier). (D) Schematic diagram of the manufacturing process of the CASR-graphene platform, integrating complementary asymmetric split-ring (CASR) metasurfaces with graphene for THz-based biosensing. (E) Effective transmission area (Δ*S*/*S*_0_) as a function of target DNA concentration, demonstrating a monotonic response with a detection limit as low as 100 nM. (F) Specificity analysis highlighting the platform’s superior selectivity for the Eae gene sequences against mismatched and nonspecific DNA sequences [106] (Copyright 2021, Elsevier). (G) Fabrication process and unit cell structure of a metasurface based on QBIC resonators with graphene (MQG) biosensor for ultrasensitive THz liquid sensing. (H) Transmission spectra of the MQG biosensor for various concentrations of target analytes [111] (Copyright 2023, John Wiley and Sons). (I) Schematic of the hybrid graphene-metallic metasurface structure designed for MIR biosensing. (J) Conceptual illustration of the sensor mechanism, showing the boronic acid-glucose binding process contributing to plasmonic resonance shifts. (K) Spectral measurements of glucose concentrations ranging from 2 nM to 20 mM, showing resonance shifts corresponding to varying concentrations. (L) Reversible glucose detection demonstrated by sequential rinsing with phosphate-buffered saline (PBS) and glucose solutions, showing the sensor’s stability and reusability [112] (Copyright 2018, Springer Nature). (M) Schematic of the active metasurface sensor with a tunable graphene Fermi level. (N) Relative absorptance spectra of the Amide I and II bands at varying gate voltages. (O) Second derivative analysis of the Amide I absorptance spectrum from human antibody IgG, showing its decomposition into secondary structural components using metasurface-enhanced spectroscopy. (P) Comparative analysis of IgG secondary structures using metasurface-enhanced FTIR and standard ATR-FTIR [113] (Copyright 2019, American Chemical Society).

One representative strategy integrates nanoslot resonators with graphene to achieve highly sensitive THz biosensing [105]. In this configuration, the nanoslots localize the electromagnetic fields and amplify the transmittance modulation (Δ*T*) induced by graphene’s conductivity tuning. Experimental results demonstrated selective and quantitative detection of DNA bases – cytosine, guanine, adenine, and thymine – with cytosine exhibiting the largest transmittance change (Δ*T*/*T*_0_ ≈ 0.24, Figure 6B). The system further enables real-time monitoring, achieving Δ*T*/*T*_0_ ≈ 0.6 after 6 h of incubation at a DNA surface density of approximately 5 nmol mm^−2^ (Figure 6C). Another approach employs complementary asymmetric split-ring (CASR) metasurfaces integrated with graphene to form a THz microfluidic biosensing platform [106]. Here, electrical tuning of graphene’s Fermi level modulates the THz transmission spectrum, enabling detection of DNA concentrations down to 100 nM and antibiotics at 0.1 mg L^−1^. The platform demonstrates high selectivity, exhibiting negligible response to nontarget sequences (Figure 6F). Further advancement has been made by incorporating graphene into THz metasurfaces supporting quasi-bound states in the continuum (qBIC, [111]). In this design, the tunability of graphene’s conductivity and its molecular adsorption capabilities enhance light–matter interaction, enabling detection of environmental toxins and biomolecules, such as APA, chlorpyrifos, and casein (Figure 6G and H). In the MIR regime, hybrid metasurfaces combining metallic nanoantennas with graphene have demonstrated ultrasensitive detection of small molecules [112]. The interaction between analytes and graphene modifies its carrier density, leading to a shift in plasmonic resonance. This approach has been utilized to detect glucose at concentrations as low as 200 pM, with a broad dynamic range up to 20 mM (Figure 6I–L). Additionally, graphene–gold hybrid metasurfaces have been engineered to enhance vibrational signatures of proteins, particularly in the Amide I and II bands [113]. Dynamic modulation of graphene’s Fermi level amplifies vibrational absorption peaks, allowing structural analysis of proteins with minimal sample volumes and sensitivity comparable to conventional Fourier-transform infrared spectroscopy (Figure 6M–P).

### 4.3 Graphene-metasurface integrated photodetector

Graphene’s high carrier mobility, broadband optical absorption, and tunable Fermi level, combined with metasurface design flexibility, make graphene-metasurface photodetectors promising for advanced photodetection applications. Graphene’s unique linear dispersion and high carrier mobility – (up to 200,000 cm^2^/Vs at room temperature) enable the efficient and ultrafast conversion of optical signals into electrical outputs, making it particularly well-suited for high-speed data communications. Additionally, graphene’s broadband spectral absorption, extending from the NIR to MIR, allows a single device to operate across multiple wavelength bands. When integrated with anisotropic metamaterial architectures, graphene can induce pronounced polarization-dependent optical responses, thereby enabling the precise detection of arbitrary polarization states. Furthermore, its Fermi level can be electrically tuned to reconfigure both optical and electrical response, providing multifunctional capabilities and low dark current for an enhanced signal-to-noise ratio. Graphene-based devices are also compatible with standard CMOS processes, paving the way for large-scale fabrication and commercialization, while the inherent flexibility and transparency of graphene support innovative metasurface designs and flexible electronics. By harnessing these distinctive attributes, graphene-based metasurface photodetectors can achieve ultrahigh-speed data transmission, broad spectral coverage, and advanced polarization control, solidifying their position as core components for next-generation optoelectronic systems.

**Figure 7:**
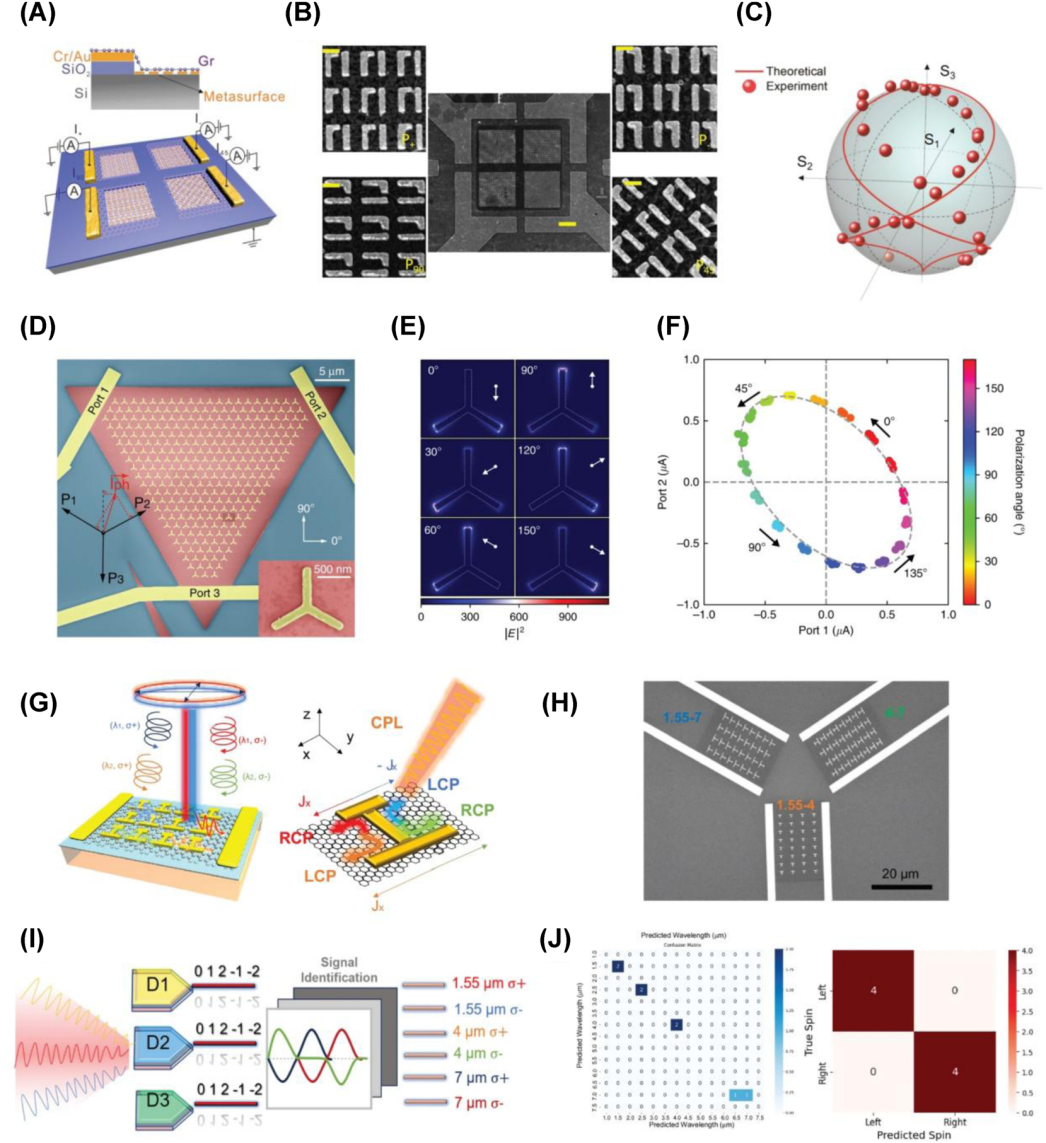
Graphene-based metasurface photodetectors: advancing multidimensional optical signal detection. (A) Schematic illustration of a graphene–silicon hybrid photodetector integrated with chiral plasmonic metasurfaces. (B) Scanning electron microscopy (SEM) image of the hybrid photodetector. (C) Measured Stokes parameters plotted on a Poincaré sphere for elliptically polarized incident light. Confirmed the device’s capability to accurately reconstruct the full-Stokes polarization state [114] (Copyright 2020, American Chemical Society). (D) Schematic of a three-port graphene photodetector designed for calibration-free polarization angle detection. (E) Simulated near-field distribution of the metasurface depending on the polarization angles. (F) Measured photocurrents at two ports plotted as a 2D closed curve, demonstrating the separation of light intensity and polarization [23] (Copyright 2020, Springer Nature). (G) Schematic of the three-port metasurface system designed for multidimensional optical detection. (H) Scanning electron microscope (SEM) image of the three-port graphene device. (I) Schematic representation of the signal extraction process for mixed light incidence. Photovoltage signal encoding enables the extraction of both wavelength and circular polarization information from the incident light. (J) Confusion matrix illustrating the performance of the machine learning model in predicting wavelength (left) and spin information (right). The high accuracy along the diagonal demonstrates the model’s precision in multidimensional optical detection [116] (Copyright 2024, Springer Nature).

An exemplary study [25] reported the development of a high-speed graphene-based photodetector, achieving a bandwidth exceeding 500 GHz and supporting data transmission rates of up to 132 Gbps. The device architecture incorporates a metamaterial perfect absorber composed of a gold reflector, an Al_2_O_3_ spacer, a monolayer graphene layer, and metallic resonators, optimized for broadband operation over a spectral range of 1,400–4,200 nm. This structure enables near-perfect absorption and efficient carrier extraction, resulting in an external responsivity of 0.75 mA/W and an internal responsivity of 1.57 mA/W at 1,550 nm, with linear operation maintained up to an input power of 100 mW. Expanding the functional scope, a recent study [114] introduces a graphene–silicon hybrid photodetector integrated with chiral plasmonic metasurfaces to enable full-Stokes polarization analysis at NIR wavelengths (1,550 nm, Figure 7A–C). In this device, graphene forms a Schottky junction with silicon, reducing dark current and thereby enhancing the signal-to-noise ratio. The photodetector comprises four distinct detection regions, each containing a chiral plasmonic metasurface designed to respond to a particular polarization component. By individually detecting the photocurrent from each region, the full set of Stokes parameters (*S*_0_, *S*_1_, *S*_2_, and *S*_3_) can be retrieved, enabling a complete characterization of the incident light’s polarization state. In contrast to multiregion architectures, Wei et al. [23] employ a single, noncentrosymmetric metasurface to determine polarization angles in the MIR range (4–10 μm) without external calibration (Figure 7D–F). The asymmetric metasurface configuration leverages the bulk photovoltaic effect (BPVE) to generate zero-bias directional photocurrents under uniform illumination. In this configuration, both the magnitude and direction of the photocurrent are governed by the polarization angle, enabling direct readout of the polarization angle. The device attains a responsivity of up to 0.12 mA/W at room temperature, three orders of magnitude higher than conventional BPVE-based detectors, while eliminating the need for any external reference. Expanding on this concept, the same group further proposed graphene-based photodetectors operating at a wavelength of 4 μm to directly detect of circularly polarized light (CPL) without the need for additional wave plates or polarizers [115]. By incorporating plasmonic nanostructures that exploit hot-electron processes at the graphene–metal interface, the mirror-symmetric metasurface design remains insensitive to both unpolarized and linearly polarized light. The mirror-symmetric metasurface yields handedness-specific directional photocurrents while maintaining insensitivity to other polarization states, underscoring the device’s CPL-focused functionality. This design achieves a high responsivity of 392 V/W at zero bias, demonstrating excellent precision in CPL detection. Extending the concept of multiparameter detection, Jiang et al. [116] introduced a graphene-based multiport metasurface system capable of simultaneously acquiring multidimensional optical information, including polarization states and wavelength components (Figure 7G–J). Operating over a broad spectral range (1–8 μm), this system achieves a wavelength prediction accuracy within 0.5 μm. Its three-port metasurface design routes distinct spectral components into separate channels, enabling concurrent segmentation and extraction of both polarization and spectral features. Photocurrents obtained from the different ports are analyzed using machine learning algorithms, resulting in a responsivity of up to 0.42 A/W at 1.55 μm and a signal-to-noise ratio exceeding 38 dB. This combination of multiport metasurface engineering with computational data processing leads to robust and accurate detection of complex optical signals across broad spectral domains. A comparative summary of these representative graphene-based metasurface photodetector designs, highlighting their working principles, metasurface configurations, responsivity metrics, and spectral operating ranges, is provided in Table 1.

**Table 1:** Comparison of diverse graphene-based metasurface photodetector designs spanning NIR to MIR wavelengths.

Paper	Principle	Metasurface design	Responsivity (units)	Range (μm)	Key features
[114]	Full-Stokes polarimetry with multiple metasurfaces	Four chiral metasurfaces	N/A	1.55	Full-Stokes analysis, polarization sensitivity
[23]	Zero-bias polarization angle detection using BPVE	Noncentrosymmetric nanoantennas	0.12 (A/W)	4–10	Calibration-free, zero-bias operation
[25]	High-speed operation with metamaterial perfect absorbers	Metamaterial perfect absorber	0.75 (A/W external), 1.57 (A/W internal)	1.4–4.2	Ultra-high bandwidth (>500 GHz)
[116]	Multidimensional detection (polarization and wavelength) with machine learning	Cooperative metasurfaces for polarization and wavelength	0.42 (mA/W)	1–8	Simultaneous polarization and wavelength detection
[22]	Enhanced photocarrier trapping with 2D slit-induced potential wells	2D dielectric slit structures	0.2–38 (A/M)	1.55–11	Broadband, CMOS compatibility
[115]	CPL-specific detection with vectorial photoresponse	Mirror-symmetric plasmonic nanostructures	392 (V/M)	4	Infinite discrimination ratio, immunity to linear/unpolarized light

## 5 Fabrication challenges for graphene-based metasurface

Among the key determinants of gate-tunable graphene-based metasurface performance are the quality of graphene – such as carrier mobility, intrinsic doping, uniformity, and sample size – and the gating strategy employed. The functional behavior of such metasurfaces is highly sensitive to the structural integrity and cleanliness of the graphene layer, both of which are governed by the synthesis and transfer processes.

While mechanically exfoliated graphene offers near-ideal optical and electronic properties, its scalability remains inherently limited. Consequently, chemical vapor deposition (CVD, [4], [117], [118]) has emerged as a viable alternative, enabling wafer-scale growth of reasonably high-quality monolayer graphene. Although CVD on metal foils – particularly copper – is the most widely adopted technique due to its scalability and cost-effectiveness, the resulting graphene films often exhibit various imperfections, including high nucleation density, polycrystallinity, surface contamination, adlayers, and mechanical deformations such as folds and wrinkles. From the perspective of commercial deployment of graphene-based metasurfaces, particularly for optoelectronic and photonic applications, the ability to reliably synthesize wafer-scale graphene with uniform and stable optical properties is of paramount importance. For transfer-oriented graphene synthesis, the major challenges stem from intrinsic stress-induced wrinkles due to thermal mismatch with metallic substrates, grain boundaries originating from polycrystalline Cu foils, and uncontrolled multilayer formation – all of which deteriorate the uniformity and quality of the transferred graphene films. In addition, contamination and defect formation arising from the metal substrates and carbon precursors further complicate the transfer process and compromise the reproducibility of device fabrication. These structural imperfections critically undermine the optical and electronic performance of graphene-based metasurfaces, rendering the deterministic growth of large-area, single-crystal, and defect-free graphene an essential milestone for their practical application.

Among various efforts to overcome these challenges, most strategies ultimately converge on substrate engineering [4], [118] – the deliberate design and modification of the substrate’s composition [4], [7], crystallography [3], [6], [119], and surface morphology [120] – to enhance the quality, uniformity, and controllability of graphene growth. Beyond the growth process, an additional challenge lies in transferring the synthesized graphene onto target substrates without compromising its intrinsic properties [121], [122], [123], [124], [125], [126], [127]. Conventional PMMA-based wet transfer techniques, widely adopted for CVD-grown graphene, often introduce structural defects such as cracks, wrinkles, and folds, along with surface contamination by polymer residues. To address these issues, various approaches have been developed, including the use of alternative transfer media (e.g., ethylene-vinyl acetate [128], paraffin [128], [129]) that reduce residue formation and mechanical damage, as well as the introduction of a sacrificial self-release layer between the elastomer stamp and graphene, which facilitates residue-free and high-fidelity transfer onto soft and fragile surfaces [130].

More recently, advanced transfer strategies have been proposed to further enhance film quality and scalability [126], [127], [131]. These include thermally driven conformal transfer techniques – employing volatile additives or low-Tg polymers embedded in PMMA matrices [112] – as well as dry-transfer methods utilizing UV-responsive adhesive tapes with tunable adhesion [132], [133]. These approaches have demonstrated significant improvements in transfer yield, film uniformity, and electronic performance, while minimizing contamination and mechanical stress. Such progress is critical to enable the reliable integration of high-quality, large-area graphene into optoelectronic and photonic metasurfaces.

Once high-quality graphene is successfully integrated onto the substrate, the design of the gating structure becomes equally vital. Effective electrostatic modulation of the graphene layer requires a combination of high capacitance and minimal optical interference. Various dielectric materials such as SiO_2_, Al_2_O_3_, and ionic gels [54], [55], [134], [135] have been utilized, each offering different advantages in terms of dielectric strength, frequency response, and compatibility with device architectures. In particular, ionic-liquid and gel-based dielectrics are favored in side-gate configurations due to their high capacitance and low voltage requirements. Three primary gate geometries have been developed: top-gate, back-gate, and side-gate configurations. In the top-gate setup, the gate electrode lies above the graphene layer, separated by a thin dielectric. To avoid obstructing the propagation of incident electromagnetic waves, grating-patterned top electrodes have been employed, although polarization sensitivity remains a concern. In contrast, the back-gate structure utilizes the substrate itself as the dielectric, offering minimal optical interference and greater design simplicity. The side-gate configuration, often paired with ionic-liquid or gel dielectrics, enables high-efficiency gating with low operating voltages and minimal impact on wave transmission.

## 6 Outlook for graphene-based metasurface

### 6.1 Toward all-optical modulation

Drawing from the widely used techniques such as pump-probe spectroscopy and high-field THz spectroscopy, which are commonly employed to study carrier dynamics in nanomaterials, including 2D materials, all-optical active modulation has been achieved through mechanisms like Pauli blocking and optical doping. Accordingly, optical pump-probe spectroscopy, employing various combinations of pump/probe frequencies, has been applied to investigate the transient responses in both interband and intraband transitions in graphene. When ultra-short laser pulses are absorbed by graphene, they induce photoexcitation of charge carriers, initiating a complex thermalization process. In this section, we synthesize the insights gained from both optical pump-driven and THz field-induced modulation in graphene and graphene-based metasurfaces, discussing how these advanced approaches could be integrated to realize even more sophisticated, multifunctional photonic systems. By combining the ultrafast [46], [136], [137], nonlinear [138], [139] and dynamically tunable properties with precisely engineered metasurface designs, future research holds the potential to unlock entirely new regimes in high-speed optical communication, sensing, and beyond. This will lay the groundwork for the next generation of ultrafast graphene-based photonic technologies.

#### 6.1.1 Optical pump-driven modulation with graphene-integrated metasurface

Dynamic control of light is a cornerstone of modern photonics, enabling a wide range of applications in optical communication, sensing, and imaging. However, achieving ultrafast modulation across broad frequency ranges, particularly in the IR and THz domains, remains a challenge. Conventional approaches typically rely on static designs or electronic control, both of which are limited by slower response times and narrower tunability. Graphene-based metasurfaces offer a promising alternative to traditional static designs or electronic control, which have slower response times and narrower tunability. Graphene, with its broadband absorption, ultrafast carrier dynamics, and tunable conductivity, offers a promising solution for high-speed optical modulation. Its linear and gapless band structure enables strong and broadband absorption, while its femtosecond to picosecond carrier relaxation dynamics allow for rapid optical response. In this context, graphene’s exceptional optical properties such as broadband absorption, ultrafast carrier dynamics, and tunable conductivity present a transformative opportunity for optical modulation. Its linear and gapless band structure allows for broadband absorption, while its ultrafast carrier relaxation time (on the order of femtoseconds to picoseconds) enables rapid responses to optical excitation. Experimental setups for graphene-based optical modulation often involve pump-probe techniques, in which femtosecond optical pulses excite the graphene layer, inducing ultrafast carrier dynamics [46], [140], [141], [142], [143], [144], [145]. Probes across various frequency ranges then measure the resulting modulation in transmission, reflection, or absorption over time scales ranging from fs to ps.

**Figure 8:**
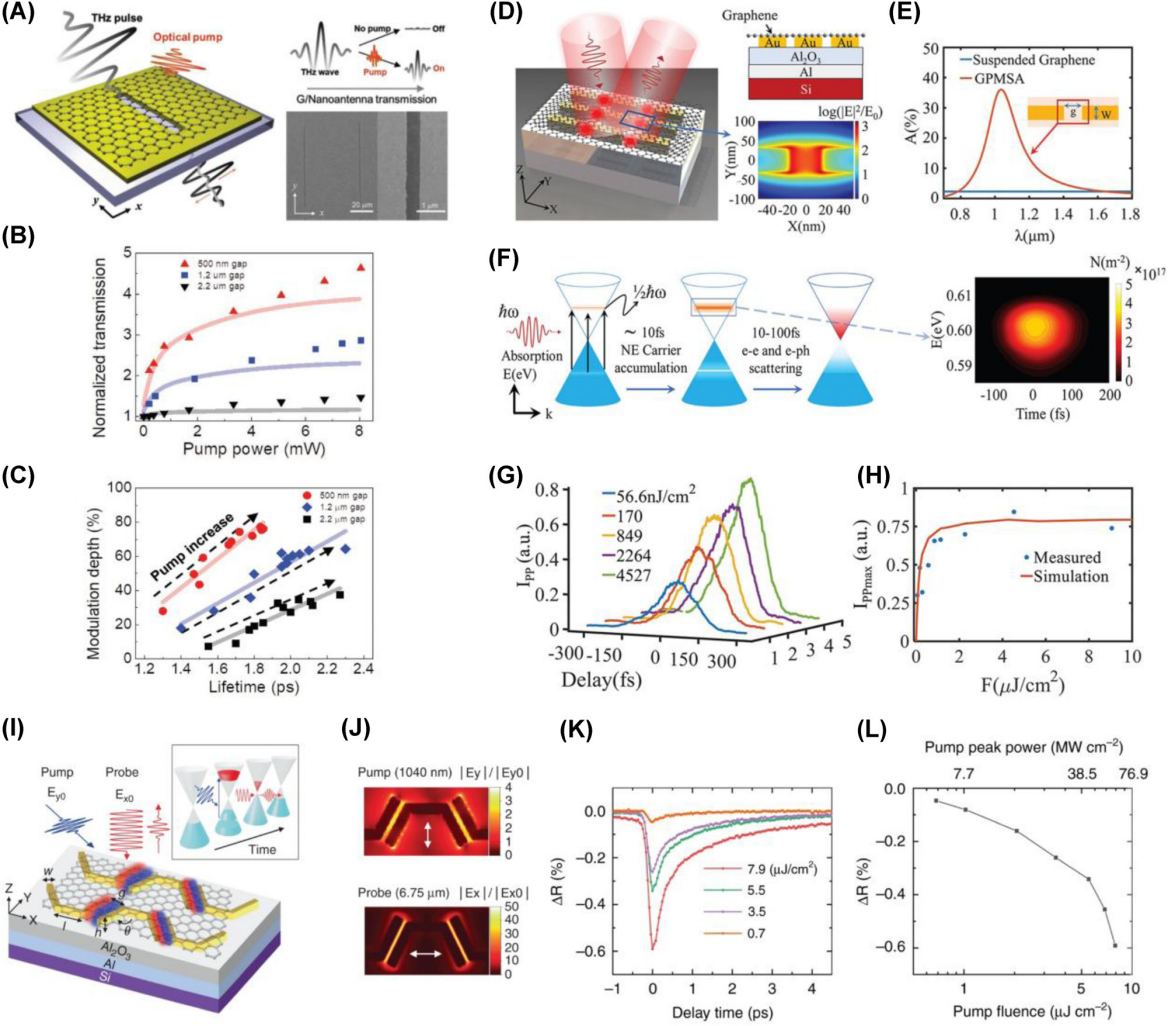
Ultrafast optical modulation. (A) Schematic diagram of the graphene–metal nanoslot antenna metasurface designed for ultrafast THz modulation. (B) Normalized transmission at the resonant frequency as a function of optical pump power for various gap sizes. (C) Summary of THz modulation depth as a function of carrier lifetime [146] (Copyright 2021, John Wiley and Sons). (D) Schematic representation of the graphene-plasmonic hybrid metasurface saturable absorber. (E) Simulation results illustrating enhanced light absorption in the nanogap regions. (F) Carrier dynamics under femtosecond laser excitation: nonequilibrium carrier generation and relaxation processes leading to a hot Fermi–Dirac distribution (left). Simulated 2D contour plot of the photoexcited nonequilibrium electron distribution (right). (G) Measured pump-probe signals at varying pump fluences. (H) Measured and simulated pump-probe signal peaks with respect to incident pump fluences [147] (Copyright 2023, American Chemical Society). (I) Schematic of the graphene–metal hybrid metasurface for broadband infrared modulation. (J) Simulated near-field intensity enhancement in the nanogap regions at the pump wavelength of 1,040 nm (top) and the probe wavelength of ∼6.5 μm (bottom). (K) Measured differential reflection spectra at varying pump fluences. (L) Extracted biexponential decay times from pump-probe measurements [148] (Copyright 2022, Springer Nature).

To address the challenge of achieving ultrafast and efficient THz modulation, Choi et al. [146] developed a graphene–metal metasurface that leverages the unique carrier dynamics of graphene. The nanoslot antenna array, integrated with graphene, enhanced localized field interactions, enabling precise and high-speed THz modulation (Figure 8A). Optical pumping with a 790 nm fs laser transiently altered the carrier scattering time in graphene, impacting the THz transmission properties. Experimental results revealed a peak modulation depth of 80 % at ∼0.9 THz, with ultrafast response times of less than 2 ps (Figure 8B and C). This work demonstrates the feasibility of graphene-based metasurfaces for noncontact, ultrafast THz applications, showcasing graphene’s unparalleled potential for next-generation communication systems. Building on graphene’s nonlinearity, Rafique et al. [147] addressed the limitations of traditional saturable absorbers in achieving low saturation fluence and ultrafast response times. The graphene-plasmonic hybrid metasurface, consisting of gold nanobars coupled with a graphene layer, created nanoscale plasmonic hotspots (Figure 8D–H) that drastically improved light–matter interactions. Optical pumping at 1.035 μm induced saturable absorption with a saturation fluence of ∼100 nJ/cm^2^, more than three orders of magnitude lower than conventional absorbers. Pump-probe dynamics further revealed an ultrafast recovery time of less than 60 fs (Figure 8G), making the device highly suitable for mode-locked lasers and high-speed optical signal processing. This work highlights how advanced hybrid metasurfaces, by strategically integrating graphene and plasmonic nanostructures, can redefine optical modulation performance. Expanding the scope of broadband optical modulation and overcoming pump efficiency limitations, Basiri et al. [148] explored graphene–metal hybrid metasurfaces for applications in the NIR and MIR regions. As shown in Figure 8I, the graphene–metal hybrid structure, enhanced by a metallic back reflector and plasmonic nanoantennas, created nanoscale hotspots that improved light–graphene interactions. Reflectance modulation results revealed substantial changes in the MIR region under optical pumping at 1.04 μm, with a reduced pump fluence of less than 70 μJ/cm^2^ (Figure 8J). Additionally, the pump-probe dynamics, shown in Figure 8K and L, demonstrated ultrafast response times of ∼2 ps. This dual-wavelength modulation design highlights graphene’s potential for ultrafast and efficient optical modulation across the infrared spectrum.

Theoretical studies have revealed graphene’s potential for advanced optical modulation by leveraging hot electron effects, quasi-Fermi level tuning, and nonlinear optical responses. One study [149] demonstrated broadband chirality control in the IR and MIR ranges using nanostructured graphene and localized surface plasmons (LSPs). Another investigation [150] showed that graphene-loaded metasurfaces enable ultrafast polarization switching with response times as fast as 200 fs, driven by hot-electron dynamics. Additionally, photoexcited graphene integrated with split-ring resonator (SRR) metasurfaces was found to amplify and tune magnetic resonances in the THz and IR ranges through quasi-Fermi level modulation [151]. These findings highlight graphene’s versatility in dynamic light manipulation, offering transformative applications in chirality control, polarization conversion, and tunable magnetic resonances. Together, they position graphene as a key material for broadband ultrafast optical modulation.

#### 6.1.2 THz field-induced nonlinear modulation in graphene and graphene-based metasurface

The increasing demand for ultrafast and efficient THz signal processing has brought nonlinear photonic materials to the forefront of research. Among these, graphene stands out as a prime candidate due to its exceptional carrier dynamics, tunable optical conductivity, and intrinsic symmetry-breaking properties. Its unique band structure and ultrafast relaxation times enable efficient high-harmonic generation (HHG) and other nonlinear THz applications, establishing graphene as a transformative platform in nonlinear photonics [139], [152].

**Figure 9:**
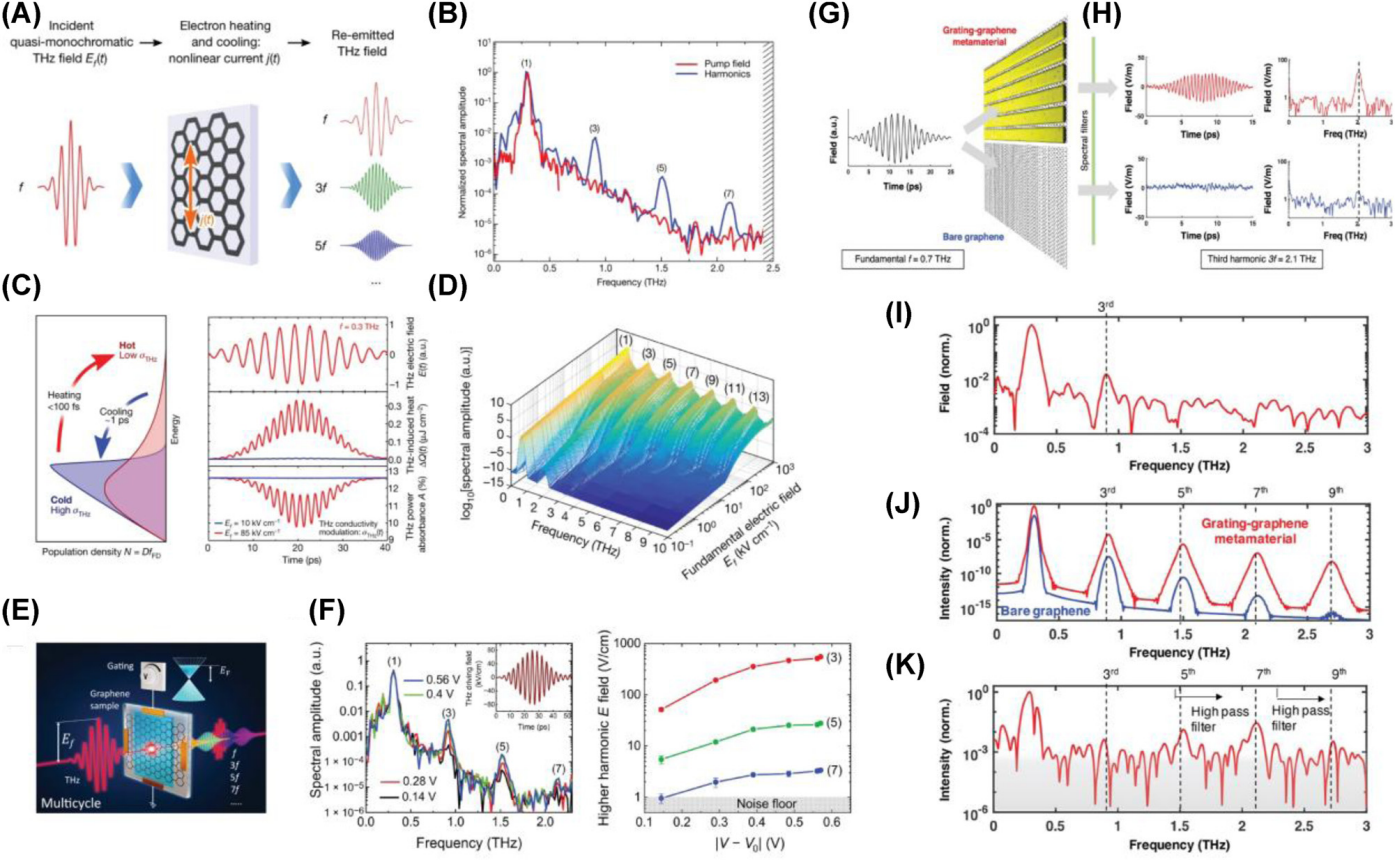
Nonlinearity: (A) schematic representation illustrating the nonlinear response of graphene under THz fields. (B) Normalized spectral amplitudes of the generated harmonics compared to the pump field, demonstrating harmonic generation up to the seventh order. (C) Energy distribution diagram illustrating the heating and cooling processes of Dirac electron populations under THz excitation (left) and time-domain profiles illustrating conductivity modulation and THz field interactions (right). (D) Amplitude spectra of the THz field transmitted through the graphene sample, calculated using the thermodynamic mechanism, showcasing harmonic generation extending up to the 13th order [153] (Copyright 2018, Springer Nature). (E) Enhancement of third-harmonic generation (THG) efficiency in gated graphene, as shown in normalized amplitude spectra. (F) THz amplitude spectra of the incident driving field and the transmitted fields through the graphene sample (left) and peak electric field of the generated harmonics as a function of the gating voltage (right) [154] (Copyright 2021, The American Association for the Advancement of Science). (G) Schematic illustration of the grating-graphene metamaterial measurement configuration. (H) Time and frequency domain measurements of the THz field strength for the grating-graphene metamaterial (top) and bare graphene (bottom). (I) THz field spectra from the grating-graphene metamaterial, highlighting the generation of odd-order harmonics up to the ninth order. (J) Simulated harmonic intensity enhancement due to the grating structure, showing greater than seven orders of magnitude increase for higher-order harmonics compared to bare graphene. (K) Experimental spectra demonstrating the presence of higher harmonics, including fifth, seventh, and ninth, using high-pass filtering techniques to suppress lower harmonics [155] (Copyright 2020, American Chemical Society).

Graphene’s intrinsic nonlinear response under THz fields is driven by the dynamic behavior of hot Dirac fermions and their ultrafast scattering processes, as demonstrated by Hafez et al. [153]. As depicted in Figure 9A and B, exposure to multicycle THz pulses generates harmonics up to the seventh order, with efficiencies of 10^−3^, 10^−4^, and 10^−5^ for the third, fifth, and seventh harmonics, respectively. This response originates from the distinct timescales of electron–electron (e–e) scattering, which facilitates rapid thermalization into a hot Fermi–Dirac distribution, and electron–phonon (e-phonon) scattering, which dissipates energy more slowly, maintaining the carriers in a hot dynamic state. This imbalance between energy redistribution and loss creates a dynamic modulation of conductivity, as illustrated in Figure 9C, which underpins graphene’s robust and scalable response to intense THz fields. Numerical simulations in Figure 9D further demonstrate that higher field strengths can extend harmonic generation to the 13th order, highlighting graphene’s scalability for nonlinear THz applications. To further enhance graphene’s nonlinear response, electrical gating has been employed to dynamically tune its Fermi level, offering precise control over carrier density. As demonstrated by Kovalev et al. [154]. As shown in Figure 9E and F, gating enhances third-harmonic generation (THG) efficiency by two orders of magnitude, optimizing intraband conductivity and intensifying nonlinear interactions under THz fields. These results demonstrate how gating enables adaptive and programmable nonlinear responses over a range of field strengths and frequencies, showcasing the potential of gated graphene for efficient and tunable THz photonics. The combination of graphene with grating-based metasurfaces, as explored by Deinert et al. [155], further amplifies its nonlinear capabilities by enhancing local field interactions. The grating structure, depicted in Figure 9G, concentrates THz fields into nanoscale hotspots, boosting harmonic generation, as shown in Figure 9H. This integration achieves THG efficiencies of up to 1 %, as shown in Figure 9I–K, and supports harmonics as high as the ninth order. The synergy between graphene’s nonlinearity and the grating’s field-confinement properties highlights the potential of hybrid designs to overcome the limitations of standalone materials.

These results show that graphene-based metasurfaces can drive a paradigm shift in ultrafast optics, enabling precise control of light–matter interactions at extreme temporal and spatial scales. Their ability to operate on ultrafast timescales allows for the modulation of optical signals, crucial for technologies like high-speed LiDAR, quantum information processing, and real-time imaging systems. Additionally, metasurfaces integrate rapid state-switching with compact, scalable designs, unlocking new possibilities for multifunctional devices. By offering unprecedented control over light propagation and interactions, they expand our understanding of photonic processes and pave the way for breakthroughs in communication, sensing, and imaging technologies.

### 6.2 New design strategy for graphene-based metasurface

#### 6.2.1 Non-Hermitian approaches for graphene-based metasurface

**Figure 10:**
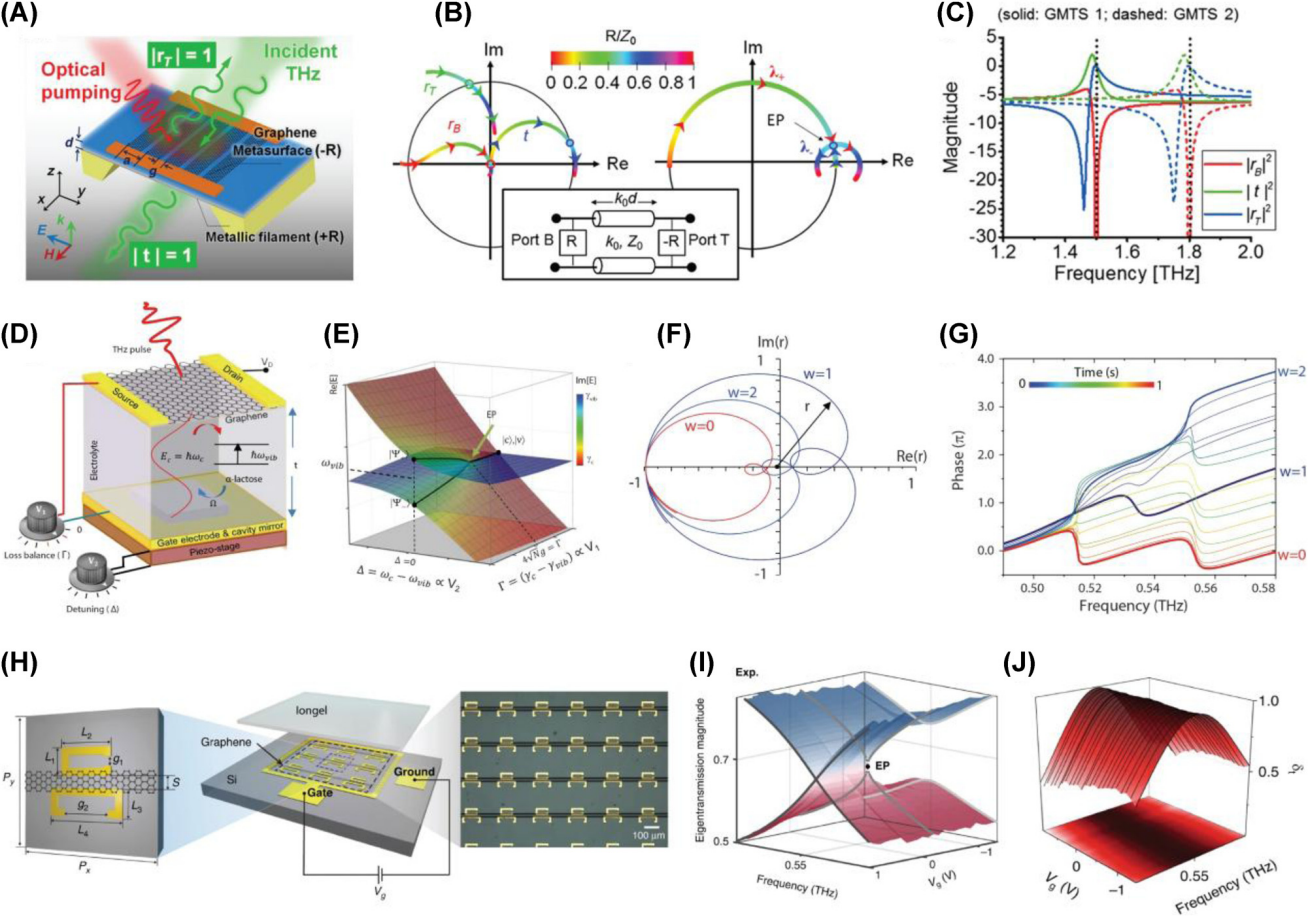
Non-Hermitian approach. (A) Schematic integration of a PT-symmetric THz system consisting of an active graphene metasurface and an absorbing metallic sheet. (B) Polar plot of scattering matrix eigenvalues, illustrating the transition from the PT-symmetric phase (left) to the symmetry-broken phase (right) as coupling strength increases. (C) Magnitude of reflectances and transmittance against the frequency for a PT-symmetric THz device based on the photoexcited graphene metasurface [182] (Copyright 2016, American Physical Society). (D) Schematic of the graphene THz resonator with *α*-lactose molecules, enabling electrical tuning of loss imbalance and frequency detuning. (E) Riemann surface of the complex eigenvalues, showing the emergence of EPs as gate voltages are varied. (F) Smith chart representation of the Fresnel reflection coefficient trajectories calculated for winding numbers *W* = 0, 2, and 1, corresponding to phase accumulations of 0, 2*π*, and 4*π*, respectively. The complex reflection coefficient *r* is plotted along the real (Re) and imaginary (Im) axes. (G) Variation of the phase of the reflected THz pulse at different time delays after applying the gate voltage [183] (Copyright 2022, The American Association for the Advancement of Science). (H) Schematic rendering and microscopic image of the non-Hermitian gated graphene-based metasurface. (I) Experimentally extracted eigentransmission Riemann surface. (J) Quantitative manifestation of asymmetric polarization conversion, showing maximal conversion at the chiral exceptional point (EP) [172] (Copyright 2023, Springer Nature).

Non-Hermitian photonic systems, which incorporate balanced gain and loss, extend beyond the scope of conventional Hermitian physics [156], [157], [158], [159], [160], [161], [162], [163]. In these systems, exceptional points (EPs) arise as unique spectral singularities where not only the eigenvalues but also the corresponding eigenstates coalesce. This reduction in the dimensionality of the eigenspace enables remarkable phenomena such as unidirectional reflection [164], [165] and heightened sensitivity [166], [167], [168], thereby opening new avenues of optical manipulation not attainable in standard Hermitian environments. Recent efforts to implement non-Hermitian physics have increasingly focused on utilizing metasurfaces as versatile platforms for engineering loss in subwavelength structure [169], [170], [171], [172], [173], [174], [175], [176], [177], [178], [179], [180], [181]. In this section, we discuss how graphene is utilized to realize and exploit EPs in non-Hermitian graphene-based metasurfaces. In a pioneering study [182], graphene played a central role in realizing a parity-time (PT) symmetric configuration that enabled unidirectional, reflectionless propagation of THz waves. By optically pumping a graphene metasurface to achieve population inversion, Chen et al. introduced negative dynamic surface resistance, which balanced the intrinsic losses of a resistive metallic filament (Figure 10A and B). To fulfill the PT symmetry condition, the graphene metasurface was patterned into nanoribbons, thus fine-tuning its surface impedance and mitigating inherent inductive behavior. Under these carefully orchestrated conditions, a spectral singularity emerged at an EP, ensuring zero reflection from one direction and finite reflection from the other (Figure 10C). In another investigation, Ergoktas et al. [183] employed electrically tunable THz resonators to explore non-Hermitian behavior in coupled light–matter systems (Figure 10D). Here, the Hamiltonian described the interaction between a THz cavity mode and the collective vibrational response of *α*-lactose molecules. Integrating a gate-tunable graphene layer made it possible to precisely control loss imbalance and frequency detuning, leading to the emergence of EPs and unveiling a complex eigenvalue landscape as shown in Figure 10E. By adjusting the gate voltage to tune optical conductivity of graphene, the system could be driven from weak to strong coupling regimes. During this process, the winding number evolved from 0 to 2, corresponding to a total phase accumulation ranging from 0 to 4*π*, thereby demonstrating the versatility of graphene in dynamically shaping the interplay between photonic and molecular modes (Figure 10F and G). In a further effort, Baek et al. [172] utilized two dipole modes to modulate the Fermi level of graphene, granting active control over the polarization state of light and defining a non-Hermitian Jones matrix for the system. By electrically gating graphene micro-ribbons, the system approached the EP, causing the eigenpolarization space to collapse as eigenstates merged (Figure 10H and I). This dimensional reduction provided a clear criterion for achieving maximal asymmetric polarization conversion, which occurred precisely at the EP (Figure 10J). By electrically tuning to this chiral EP, the study not only demonstrated a route to extreme asymmetric polarization conversion but also revealed a topological feature associated with this extraordinary condition, underscoring graphene’s unique capacity to facilitate precise polarization manipulation in non-Hermitian photonic platforms.

#### 6.2.2 Computational approaches for inverse design of graphene-based metasurface

In contrast to conventional forward design approaches – which often rely on predefined meta-atom libraries and intuitive phase maps – the inverse design paradigm systematically determines optimal metasurface configurations to achieve desired functionalities. By integrating advanced computational optimization algorithms with graphene’s uniquely tunable conductivity, researchers can attain unprecedented control over light, enabling functionalities such as broadband absorption, precise beam steering, dynamic switching, and encrypted data transmission. Conventional metasurface engineering typically assumes weak coupling, periodic boundary conditions, and coherent illumination. However, these approximations fail to address the complexity and multifunctionality required in emerging photonic applications. Inverse design methods overcome these limitations by efficiently exploring vast, high-dimensional design spaces, leveraging optimization techniques such as neural networks, gradient-based methods, evolutionary algorithms, and adjoint-based approaches. This section introduces the optimization strategies employed in the inverse design of graphene-based metasurfaces and highlights their complementary strengths and capabilities.

**Figure 11:**
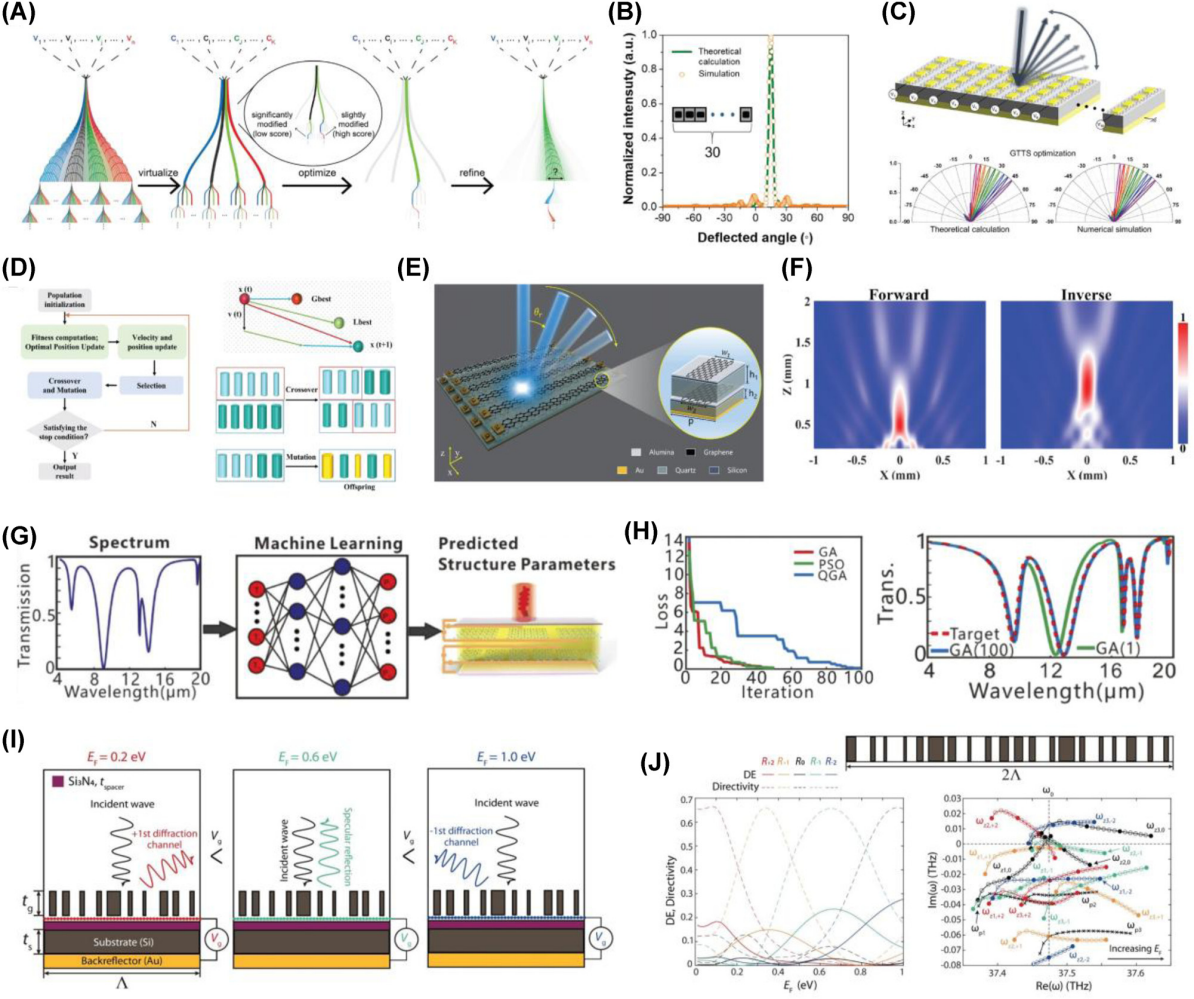
Inverse design and intelligent optimization for tunable THz wavefront control. (A) Illustration of genetic-type tree search (GTTS) algorithm for inverse design. (B) Comparison of far-field radiation intensity obtained from theoretical calculations and numerical simulations at a target angle of 15°, using the GTTS optimization approach. (C) Schematic of the beam-steering metasurface using a hybrid Au–graphene metasurface (top). Normalized far-field radiation intensity as a function of steering angle, obtained from theoretical and numerical methods, based on the Fermi energy of the Au–graphene metasurface (bottom) [184] (Copyright 2021, American Chemical Society). (D) Flowchart illustrating the steps of the genetic algorithm-particle swarm optimization (GA-PSO) hybrid optimization algorithm. (E) Schematic of the dual-parameter controlled metasurface for enhanced terahertz beamforming. (F) Comparison of simulated focusing performance between forward (left) and inverse (right) design approaches, demonstrating that inverse design leads to a more accurate focal length [185] (Copyright 2024, IOP Publishing). (G) Diagram of the design process for plasmon-induced transparency in graphene metamaterials using artificial neural networks. (H) The fitness of GA, QGA, and PSO algorithms for transmission spectrum optimization over iterations (left). Comparison of the optimized transmission spectrum (100th iteration) and the initial spectrum (1st iteration), showing closeness to the target spectrum (right) [186] (Copyright 2020, Optica Publishing Group). (I) Schematic of the three-level beam switching metasurface. The metasurface redirects incident waves into +1st, 0th, or −1st diffraction channels based on the applied gate voltage and corresponding graphene Fermi energy levels. (J) Diffraction efficiencies (DE) and directivities of the four-level beam switching metasurface for diffraction orders (left). Locations of poles (black X marks) and zeros (colored circles) as a function of graphene Fermi level (EF = 0.05–0.95 eV, step 0.05 eV). Filled circles indicate zeros at EF = 0.05, 0.35, 0.65, and 0.95 eV (right) [187] (Copyright 2024, Licensed under CC BY 4.0.).

Lin et al. [184] demonstrated an exemplary approach by employing a genetic-type tree search (GTTS) algorithm, augmented with unsupervised clustering, to design high-performance beam-steering metasurfaces (Figure 11A and B). Their methodology utilized unit cell geometry, including the dimensions and arrangement of nanoantennas, as primary design variables, alongside phase and reflectance properties derived from the optical characteristics of the structures. The GTTS algorithm iteratively refined candidate designs through selection, crossover, and mutation processes, efficiently navigating the high-dimensional design space while mitigating computational overhead through clustering. This approach facilitated the development of a hybrid Au–graphene metasurface capable of simultaneous phase modulation and reflectance control at MIR wavelengths (∼6.05 µm). The optimization target focused on maximizing directivity and achieving stable beam steering across a range of 5°–45° as shown in Figure 11C. The GTTS framework uniquely integrated clustering to group similar solutions, effectively reducing computational costs and ensuring global optimization.

Building on these advancements, Wu et al. [185] introduced a dual-parameter controlled graphene metasurface, optimized for THz frequencies, employing a hybrid genetic algorithm-particle swarm optimization (GA-PSO) approach as illustrated in Figure 11D. Unlike Lin et al.’s geometry-centric method, this work leveraged Fermi energy as a tunable parameter to dynamically control the optical properties of bilayer graphene, alongside co-optimization of phase and amplitude (Figure 11E). Independent adjustment of the Fermi energy in each graphene layer enabled 360° phase modulation and an amplitude range of 0–0.8 at 2.6 THz. The optimization targets extended beyond beam steering to include beam focusing, achieving significant enhancements in directivity (up to 60 %), focusing efficiency (from 25 % to 40 %, Figure 11F), and focusing quality (a 150 % increase). By shifting the focus to dynamic parameters and employing a dual-parameter control strategy, this study demonstrated superior multifunctional performance across a wide frequency range (1.6–5 THz). The combination of GA’s global search capabilities with PSO’s local refinement efficiency allow precise tuning of dynamic parameters, making it particularly effective for real-time applications in the THz range.

Zhang et al. [186] took a different approach by employing machine learning (ML) and evolutionary algorithms to design graphene plasmonic metamaterials, with a particular focus on plasmon-induced transparency (PIT) effects in Figure 11G. The design variables encompassed the chemical potentials of graphene ribbons (*μc*1, *μc*2, *μc*3, 4), filling ratios, and the separation between graphene layers (*dg*). The study utilized machine learning algorithms such as Random Forest (RF) and Artificial Neural Networks (ANNs), for forward spectrum prediction and inverse design. Furthermore, it employed single-objective (GA, PSO) and multi-objective (NSGA-II) optimization to achieve steep transmission characteristics for PIT. The optimization aimed to maximize the difference between transmission peaks and dips, achieving a maximum difference of 0.97 and providing critical advancements in dynamic optical modulation (Figure 11H). Unlike Lin et al. and Wu et al., Zhang et al. integrated GA with machine learning models to guide the optimization process, using ML for rapid prediction and GA for fine-tuning, creating a synergistic framework. Additionally, the use of NSGA-II enabled the balancing multiple objectives, optimizing PIT performance across several metrics simultaneously. Another study [187] demonstrated the effectiveness of adjoint-based optimization methods in engineering single-gate tunable graphene-based metasurfaces for multilevel beam switching (Figure 11I and J). The adjoint method is particularly well-suited for photonic inverse design as it efficiently computes the gradient of a figure of merit (e.g., diffraction efficiency) with respect to numerous design parameters using only two simulations – a forward simulation and an adjoint simulation. This approach reduces computational costs compared to conventional brute-force optimization, making it highly scalable for large design spaces.

In contrast to GA-based methods, which rely on iterative population-based strategies such as selection, crossover, and mutation, the adjoint method directly computes the gradient of the objective function, enabling faster and more precise optimization. Its reliance on gradient-based optimization allows for faster convergence, particularly in scenarios with well-defined physical models and continuous design spaces. In this work, the design variables included the Fermi energy of gate-tunable graphene, controlled via electrical bias, which modulated the phase and amplitude of the metasurface’s optical response. As shown in Figure 11I and J, the inclusion of a gate-tunable graphene layer enabled precise electrical modulation of diffraction efficiency in the MIR regime (∼8 µm). Using the adjoint optimization framework, the researchers efficiently identified parameter sets that achieved directivities exceeding 95 % for two-level beam switching and over 90 % for three-level configurations, while simultaneously attaining diffraction efficiencies approaching 50 %. These results highlight the computational efficiency and scalability of adjoint-based optimization methods for designing electrically reconfigurable metasurfaces, enabling precise control over beam switching and diffraction properties.

By integrating advanced inverse design techniques – such as topology optimization, evolutionary algorithms, and machine learning – with active metasurfaces, researchers can achieve dynamic and multifunctional optical control. These methods are particularly well-suited for graphene-based metasurfaces, where tunable electrical conductivity enables real-time modulation of optical properties. Applications range from dynamic beam steering and multilevel switching to broadband absorption and encrypted optical data transmission (Table 2). The synergy between advanced computational optimization techniques and graphene’s unique properties positions active metasurfaces as key components for the next generation of compact, intelligent photonic devices operating across spectral domains from the visible to THz ranges.

**Table 2:** Comparison of optimization and inverse design strategies for graphene-based metasurfaces with varying design parameters, target functionalities, and spectral ranges.

Paper	Design variables	Optimization approach	Optimization targets	Frequency range
[184]	Nanoantenna geometry (dimensions, arrangement), phase and reflectance properties	Genetic-type tree search (GTTS): clustering for efficient design space exploration	Maximize directivity and stable beam steering across 5°–45° range	MIR (∼6.05 µm)
[185]	Fermi energy for bilayer graphene; phase and amplitude co-optimization	Hybrid genetic algorithm-particle swarm optimization (GA-PSO): combines global search with rapid convergence	Beam steering and focusing: directivity (+60 %), focusing efficiency (25 % → 40 %), focusing quality (+150 %)	THz (1.6–5 THz)
[186]	Fermi energy, filling ratios, and separation	Machine learning (RF, ANN) for prediction/inverse design; combined with GA, PSO, and NSGA-II for optimization	Optimize plasmon-induced transparency (PIT) with steep transmission characteristics; peak-dip difference: 0.97	MIR (5–15 µm)
[187]	Fermi energy, structural parameters (height, thickness, position)	Adjoint-based optimization: efficient gradient computation using forward and adjoint simulations	Multi-level beam switching (2–4 levels): directivity >95 %, diffraction efficiency ∼50 %	MIR (∼8 µm)

## 7 Conclusions

Graphene-based metasurfaces have evolved from intriguing theoretical constructs into versatile, high-performance platforms for dynamic, subwavelength-scale control of electromagnetic waves. Guided by graphene’s unique electronic and optical properties – its gapless Dirac cone dispersion, exceptional carrier mobility, and tunable Fermi level – researchers have unlocked a diverse range of functionalities spanning the THz, MIR, and NIR spectral domains, extending even into the visible regime. The seamless integration of graphene’s broadband and actively tunable optical response with the design flexibility of metasurfaces has catalyzed breakthroughs in optical modulation, beam steering, polarization control, and sensing.

Central to this progress is the ability to dynamically manipulate the interplay between interband and intraband transitions in graphene through techniques such as electrical gating, optical pumping, chemical doping, and even intense THz field excitation. These innovations have given rise to novel device architectures, including hybrid graphene–metal metasurfaces for efficient mode conversion and perfect absorption, tunable quarter-wave plates in the THz regime, and advanced photodetectors capable of operating across multiple spectral bands while simultaneously analyzing polarization states. Additionally, the incorporation of non-Hermitian concepts, intelligent coding strategies, and advanced inverse design techniques – spanning machine learning to adjoint-based optimization – has expanded the design space beyond the limitations of conventional metasurface engineering.

Graphene-based metasurfaces have also demonstrated exceptional promise in biosensing, where their extreme field confinement, tunability, and label-free detection capabilities enable unprecedented sensitivity and molecular selectivity. Emerging applications, such as all-optical modulation, nonlinear THz frequency generation, and the integration of graphene with moiré lattices or van der Waals heterostructures, are paving the way for a new generation of optoelectronic and photonic devices. These advancements promise ultrafast response times, multifunctional operation, and the capacity to dynamically reconfigure optical states on demand – key features for next-generation communication systems, on-chip computing architectures, and quantum information technologies.

Looking ahead, advancements in graphene material quality, scalable production methods, and integration techniques will further enhance the performance, reproducibility, and practicality of graphene-based metasurfaces. Emerging computational tools, including artificial intelligence and topological optimization, will refine the design process, unlocking even more complex and efficient devices. As these trends converge, graphene-based metasurfaces are poised to revolutionize photonics, offering unprecedented control over light–matter interactions at the fundamental limits of space, time, and energy. In doing so, they will serve as a cornerstone for future innovations in sensing, imaging, communication, and beyond.
